# Tikhonov regularization of a second order dynamical system with Hessian driven damping

**DOI:** 10.1007/s10107-020-01528-8

**Published:** 2020-06-11

**Authors:** Radu Ioan Boţ, Ernö Robert Csetnek, Szilárd Csaba László

**Affiliations:** 1 Faculty of Mathematics, University of Vienna, Oskar-Morgenstern-Platz 1, 1090 Vienna, Austria; 2 Department of Mathematics, Technical University of Cluj-Napoca, Memorandumului 28, Cluj-Napoca, Romania

**Keywords:** Second order dynamical system, Convex optimization, Tikhonov regularization, Fast convergence methods, Hessian-driven damping, 34G25, 47J25, 47H05, 90C26, 90C30, 65K10

## Abstract

We investigate the asymptotic properties of the trajectories generated by a second-order dynamical system with Hessian driven damping and a Tikhonov regularization term in connection with the minimization of a smooth convex function in Hilbert spaces. We obtain fast convergence results for the function values along the trajectories. The Tikhonov regularization term enables the derivation of strong convergence results of the trajectory to the minimizer of the objective function of minimum norm.

## Introduction

The paper of Su et al. [20] was the starting point of intensive research of second order dynamical systems with an asymptotically vanishing damping term of the form

1 $$\begin{aligned} \ddot{x}(t)+\frac{\alpha }{t}\dot{x}(t)+{\nabla }g(x(t))=0, \ t \ge t_0 > 0, \end{aligned}$$

where $g:\mathcal {H}\longrightarrow \mathbb {R}$ is a convex and continuously Fréchet differentiable function defined on a real Hilbert space $\mathcal {H}$ fulfilling $\hbox {argmin}g \ne \emptyset$. The aim is to approach by the trajectories generated by this system the solution set of the optimization problem

2 $$\begin{aligned} \min _{x\in \mathcal {H}} g(x). \end{aligned}$$

The convergence rate of the objective function along the trajectory is in case $\alpha >3$ of

$$\begin{aligned}g(x(t))-\min g=o\left( \frac{1}{t^2}\right) ,\end{aligned}$$

while in case $\alpha =3$ it is of

$$\begin{aligned}g(x(t))-\min g=O\left( \frac{1}{t^2}\right) , \end{aligned}$$

where $\min g \in \mathbb {R}$ denotes the minimal value of *g*. Also in view of this fact, system (1) is seen as a continuous version of the celebrated Nesterov accelerated gradient scheme (see [16]). In what concerns the asymptotic properties of the generated trajectories, weak convergence to a minimizer of *g* as the time goes to infinity has been proved by Attouch et al. [7] (see also [6]) for $\alpha > 3$. Without any further geometrical assumption on *g*, the convergence of the trajectories in the case $\alpha \le 3$ is still an open problem.

Second order dynamical systems with a geometrical Hessian driven damping term have aroused the interest of the researchers, due to both their applications in optimization and mechanics and their natural relations to Newton and Levenberg-Marquardt iterative methods (see [2]). Furthermore, it has been observed for some classes of optimization problems that a geometrical damping term governed by the Hessian can induce a stabilization of the trajectories. In [11] the dynamical system with Hessian driven damping term

3 $$\begin{aligned} \ddot{x}(t)+\frac{\alpha }{t}\dot{x}(t)+\beta {\nabla }^2g(x(t))\dot{x}(t)+{\nabla }g(x(t))=0, \ t \ge t_0 > 0, \end{aligned}$$

where $\alpha \ge 3$ and $\beta >0$, has been investigated in relation with the optimization problem (2). Fast convergence rates for the values and the gradient of the objective function along the trajectories are obtained and the weak convergence of the trajectories to a minimizer of *g* is shown. We would also like to mention that iterative schemes which result via (symplectic) discretizations of dynamical systems with Hessian driven damping terms have been recently formulated and investigated from the point of view of their convergence properties in [5, 18, 19].

Another development having as a starting point (1) is the investigation of dynamical systems involving a Tikhonov regularization term. Attouch, Chbani and Riahi investigated in this context in [8] the system

4 $$\begin{aligned} \ddot{x}(t)+\frac{\alpha }{t}\dot{x}(t)+{\nabla }g(x(t))+\epsilon (t)x(t)=0, \ t \ge t_0 > 0, \end{aligned}$$

where $\alpha \ge 3$ and $\epsilon :[t_0,+\infty )\longrightarrow [0,+\infty )$. One of the main benefits of considering such a regularized dynamical system is that it generates trajectories which converge strongly to the minimum norm solution of (2). Besides that, in [8] it was proved that the fast convergence rate of the objective function values along the trajectories remains unaltered. For more insights into the role played by the Tikhonov regularization for optimization problems and, more general, for monotone inclusion problems, we refer the reader to [3, 4, 9, 15].

This being said, it is natural to investigate a second order dynamical system which combines a Hessian driven damping and a Tikhonov regularization term and to examine if it inherits the properties of the dynamical systems (3) and (4). This is the aim of the manuscript, namely the analysis in the framework of the general assumption stated below of the dynamical system

5 $$\begin{aligned} \ddot{x}(t)+\frac{\alpha }{t}\dot{x}(t)+\beta {\nabla }^2g(x(t))\dot{x}(t)+{\nabla }g(x(t))+\epsilon (t)x(t)=0, \ t \ge t_0 > 0, \ x(t_0)=u_0, \ \dot{x}(t_0)=v_0, \end{aligned}$$

where $\alpha \ge 3$ and $\beta \ge 0$, and $u_0, v_0 \in \mathcal{H}$.

**General assumption:**

- $g:\mathcal {H}\longrightarrow \mathbb {R}$ is a convex and twice Fréchet differentiable function with Lipschitz continuous gradient on bounded sets and $\hbox {argmin}g \ne \emptyset$;
- $\epsilon :[t_0,+\infty )\longrightarrow [0,+\infty )$ is a nonincreasing function of class $C^1$ fulfilling $\lim _{t\longrightarrow +\infty }\epsilon (t)=0$.

The fact that the starting time $t_0$ is taken as strictly greater than zero comes from the singularity of the damping coefficient $\frac{\alpha }{t}$. This is not a limitation of the generality of the proposed approach, since we will focus on the asymptotic behaviour of the generated trajectories. Notice that if $\mathcal {H}$ is finite-dimensional, then the Lipschitz continuity of $\nabla g$ on bounded sets follows from the continuity of $\nabla ^2 g$.

To which extent the Tikhonov regularization does influence the convergence behaviour of the trajectories generated by (5) can be seen even when minimizing a one dimensional function. Consider the convex and twice continuously differentiable function

6 $$\begin{aligned} g : \mathbb {R}\rightarrow \mathbb {R}, \quad g(x)=\left\{ \begin{array}{c@{\quad }l} -(x+1)^3, &{} \text{ if } x < -1\\ 0, &{} \text{ if } -1\le x\le 1\\ (x-1)^3, &{} \text{ if } x > 1. \end{array}\right. \end{aligned}$$

**Fig. 1:**
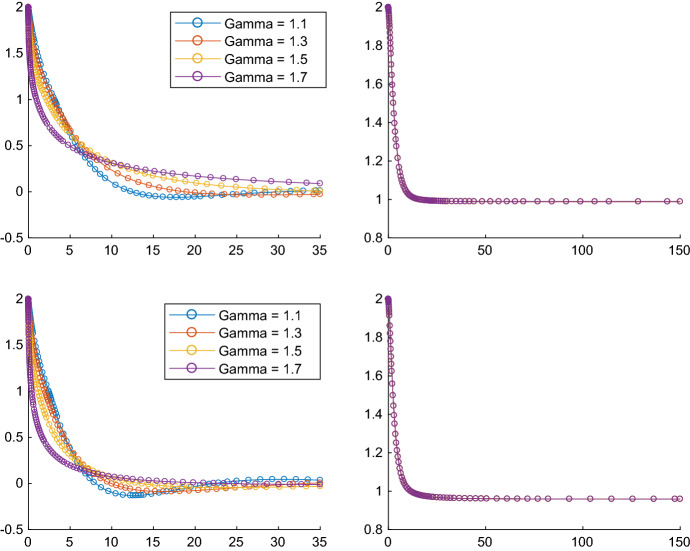
First column: the trajectories of the dynamical system with Tikhonov regularization $\epsilon (t)=t^{-\gamma }$ are approaching the minimum norm solution $x^*=0$. Second column: the trajectories of the dynamical system without Tikhonov regularization the trajectory are approaching the optimal solution 1

It holds that $\hbox {argmin}g=[-1,1]$ and $x^*=0$ is its minimum norm solution. In the second column of Fig. 1 we can see the behaviour of the trajectories generated by the dynamical system without Tikhonov regularization (which corresponds to the case when $\epsilon$ is identically 0) for $\beta =1$ and $\alpha =3$ and, respectively, $\alpha =4$. In both cases the trajectories are approaching the optimal solution 1, which is a minimizer of *g*, however, not the minimum norm solution.

In the first column of Fig. 1 we can see the behaviour of the trajectories generated by the dynamical system with Tikhonov parametrizations of the form $t \mapsto \epsilon (t)=t^{-\gamma }$, for different values of $\gamma \in (1,2)$, which is in accordance to the conditions in Theorem 4.4, $\beta =1$ and $\alpha =3$ and, respectively, $\alpha =4$. The trajectories are approaching the minimum norm solution $x^*=0$.

The organization of the paper is as follows. We start the analysis of the dynamical system (5) by proving the existence and uniqueness of a global $C^2$-solution. In the third section we provide two different settings for the Tikhonov parametrization $t \mapsto \epsilon (t)$ in both of which *g*(*x*(*t*)) converges to $\min g$, the minimal value of *g*, with a convergence rate of $O\left( \frac{1}{t^2}\right)$ for $\alpha =3$ and of $o\left( \frac{1}{t^2}\right)$ for $\alpha >3$. The proof relies on Lyapunov theory; the choice of the right energy functional plays a decisive role in this context. Weak convergence of the trajectory is also derived for $\alpha >3$. In the last section we focus on the proof of strong convergence to a minimum norm solution: firstly, in a general setting, for the ergodic trajectory, and, secondly, in a slightly restrictive setting, for the trajectory *x*(*t*) itself.

## Existence and uniqueness

In this section we will prove the existence and uniqueness of a global $C^2$-solution of the dynamical system (5). The proof of the existence and uniqueness theorem is based on the idea to reformulate (5) as a particular first order dynamical system in a suitably chosen product space (see also [11]).

### Theorem 2.1

For every initial value $(u_0, v_0) \in \mathcal {H}\times \mathcal {H}$, there exists a unique global $C^2$-solution $x:[t_0, + \infty ) \rightarrow \mathcal {H}$ to (5).

### Proof

Let $(u_0, v_0) \in \mathcal {H}\times \mathcal {H}$. First we assume that $\beta =0$, which gives the dynamical system (4) investigated in [8]. The statement follows from [14, Proposition 2.2(b)] (see also the discussion in [8, Section 2]).

Assume now that $\beta >0$. We notice that $x:[t_0,+\infty )\longrightarrow \mathcal {H}$ is a solution of the dynamical system (5), that is

$$\begin{aligned} \ddot{x}(t)+\frac{\alpha }{t}\dot{x}(t)+\beta {\nabla }^2g(x(t))\dot{x}(t)+{\nabla }g(x(t))+\epsilon (t)x(t){=}0,\,\,x(t_0){=}u_0,\,\dot{x}(t_0){=}v_0, \end{aligned}$$

if and only if $(x,y) :[t_0,+\infty )\longrightarrow \mathcal {H} \times \mathcal {H}$ is a solution of the dynamical system

$$\begin{aligned}\left\{ \begin{array}{lll} \dot{x}(t)+\beta {\nabla }g(x(t))-y(t)=0\\ \dot{y}(t)+\frac{\alpha }{t}\dot{x}(t)+{\nabla }g(x(t))+\epsilon (t)x(t)=0\\ x(t_0)=u_0,\,y(t_0)=v_0+\beta {\nabla }g(u_0), \end{array} \right. \end{aligned}$$

which is further equivalent to

7 $$\begin{aligned} \left\{ \begin{array}{lll} \dot{x}(t)+\beta {\nabla }g(x(t))-y(t)=0\\ \dot{y}(t) + \frac{\alpha }{t} y(t) + \left( 1- \frac{\alpha \beta }{t}\right) {\nabla }g(x(t)) +\epsilon (t)x(t)=0\\ x(t_0)=u_0,\,y(t_0)=v_0+\beta {\nabla }g(u_0). \end{array} \right. \end{aligned}$$

We define $F:[t_0,+\infty ) \times \mathcal {H} \times \mathcal {H} \rightarrow \mathcal {H} \times \mathcal {H}$ by

$$\begin{aligned}F(t,u,v) = \left( -\beta {\nabla }g(u) + v, - \frac{\alpha }{t} v - \left( 1- \frac{\alpha \beta }{t}\right) {\nabla }g(u) - \epsilon (t)u\right) , \end{aligned}$$

and write (7) as

8 $$\begin{aligned} \left\{ \begin{array}{lll} \big (\dot{x}(t), \dot{y}(t)\big ) = F(t,x(t),y(t))\\ \big (x(t_0), y(t_0)\big )=\big (u_0, v_0+\beta {\nabla }g(u_0)\big ). \end{array} \right. \end{aligned}$$

Since ${\nabla }g$ is Lipschitz continuous on bounded sets and continuously differentiable, the local existence and uniqueness theorem (see [17, Theorems 46.2 and 46.3]) guarantees the existence of a unique solution (*x*, *y*) of (8) defined on a maximum intervall $[t_0,T_{\max })$, where $t_0 < T_{\max }\le +\infty .$ Furthermore, either $T_{\max } = +\infty$ or $\lim _{t \rightarrow T_{\max }} \Vert x(t)\Vert + \Vert y(t)\Vert = +\infty$. We will prove that $T_{\max }=+\infty$, which will imply that *x* is the unique global $C^2$-solution of (5).

Consider the energy functional (see [10])

$$\begin{aligned}\mathcal{E} : [t_0, +\infty ) \rightarrow \mathbb {R}, \quad \mathcal{E}(t)=\frac{1}{2}\Vert \dot{x}(t)\Vert ^2+g(x(t))+\frac{1}{2}\epsilon ({t})\Vert x(t)\Vert ^2.\end{aligned}$$

By using (5) we get

$$\begin{aligned}\frac{d}{dt} \mathcal{E}(t)=-\frac{\alpha }{t}\Vert \dot{x}(t)\Vert ^2-\beta \langle {\nabla }^2 g(x(t))\dot{x}(t),\dot{x}(t)\rangle +\frac{1}{2}\dot{\epsilon }(t)\Vert x(t)\Vert ^2,\end{aligned}$$

and, since $\epsilon$ is nonincreasing and ${\nabla }^2 g(x(t))$ is positive semidefinite, we obtain that

$$\begin{aligned}\frac{d}{dt} \mathcal{E}(t) \le 0 \quad \forall t \ge t_0.\end{aligned}$$

Consequently, $\mathcal{E}$ is nonincreasing, hence

$$\begin{aligned} \frac{1}{2}\Vert \dot{x}(t)\Vert ^2+g(x(t))+\frac{1}{2}\epsilon ({t})\Vert x(t)\Vert ^2\le \frac{1}{2}\Vert \dot{x}(t_0)\Vert ^2+g(x(t_0))+\frac{1}{2}\epsilon (t_0)\Vert x(t_0)\Vert ^2 \ \quad \forall t \ge t_0. \end{aligned}$$

From the fact that *g* is bounded from below we obtain that $\dot{x}$ is bounded on $[t_0, T_{\max })$. Let $\Vert \dot{x}\Vert _\infty :=\sup _{t\in [t_0, T_{\max })}\Vert \dot{x}(t)\Vert < +\infty .$

Since $\Vert x(t)-x(t')\Vert \le \Vert \dot{x}\Vert _\infty |t-t'|$ for all $t,t' \in [t_0, T_{\max })$, there exists $\lim _{t\longrightarrow T_{\max }}x(t)$, which shows that *x* is bounded on $[t_0, T_{\max })$. Since $\dot{x}(t)+\beta {\nabla }g(x(t))=y(t)$ for all $t \in [t_0, T_{\max })$ and $\nabla g$ is Lipschitz continuous on bounded sets, it yields that *y* is also bounded on $[t_0,T_{\max })$. Hence $\lim _{t \rightarrow T_{\max }} \Vert x(t)\Vert + \Vert y(t)\Vert$ cannot be $+\infty$, thus $T_{\max }=+\infty ,$ which completes the proof. $\square$

## Asymptotic analysis

In this section we will show to which extent different assumptions we impose to the Tikhonov parametrization $t \mapsto \epsilon (t)$ influence the asymptotic behaviour of the trajectory *x* generated by the dynamical system (5). In particular, we are looking at the convergence of the function *g* along the trajectory and the weak convergence of the trajectory.

We recall that the asymptotic analysis of the system (5) is carried out in the framework of the general assumptions stated in the introduction.

We start with a result which provides a setting that guarantees the convergence of *g*(*x*(*t*)) to $\min g$ as $t \rightarrow +\infty$.

### Theorem 3.1

Let *x* be the unique global $C^2$-solution of (5). Assume that one of the following conditions is fulfilled:

- $\int _{t_0}^{+\infty }\frac{\epsilon (t)}{t}dt<+\infty$ and there exist $a > 1$ and $t_1\ge t_0$ such that
  $$\begin{aligned}\dot{\epsilon }(t)\le -\frac{a\beta }{2}\epsilon ^2(t) \text{ for } \text{ every } t\ge t_1;\end{aligned}$$
- there exists $a >0$ and $t_1\ge t_0$ such that
  $$\begin{aligned}\epsilon (t)\le \frac{a}{t} \text{ for } \text{ every } t\ge t_1. \end{aligned}$$

If $$\alpha \ge 3$$, then

$$\begin{aligned}\lim _{t \rightarrow + \infty }g(x(t))=\min g.\end{aligned}$$

### Proof

Let be $x^* \in \hbox {argmin}g$ and $2\le b\le \alpha -1$ be fixed. We introduce the following energy functional $\mathcal {E}_{b} : [t_0, +\infty ) \rightarrow \mathbb {R}$,

9 $$\begin{aligned} \mathcal {E}_{b}(t)=&\ (t^2-\beta (b+2-\alpha )t)\left( g(x(t))-\min g\right) +\frac{t^2\epsilon (t)}{2}\Vert x(t)\Vert ^2\nonumber \\&\ +\frac{1}{2}\Vert b(x(t)-x^*)+t(\dot{x}(t)+\beta {\nabla }g(x(t)))\Vert ^2+\frac{b(\alpha -1-b)}{2}\Vert x(t)-x^*\Vert ^2. \end{aligned}$$

For every $t \ge t_0$ it holds

10 $$\begin{aligned} \dot{\mathcal {E}_{b}}(t) =&\ (2t-\beta (b+2-\alpha ))\left( g(x(t))-\min g\right) \nonumber \\&\ +(t^2-\beta (b+2-\alpha )t)\langle {\nabla }g(x(t),\dot{x}(t)\rangle +\frac{t^2\dot{\epsilon }(t)+2t\epsilon (t)}{2}\Vert x(t)\Vert ^2+t^2\epsilon (t)\langle \dot{x}(t),x(t)\rangle \nonumber \\&\ +\langle (b+1)\dot{x}(t)+\beta {\nabla }g(x(t))+t(\ddot{x}(t)+\beta {\nabla }^2g(x(t))\dot{x}(t)), b(x(t)-x^*)+t(\dot{x}(t)+\beta {\nabla }g(x(t)))\rangle \nonumber \\&\ +b(\alpha -1-b)\langle \dot{x}(t),x(t)-x^*\rangle . \end{aligned}$$

Now, by using (5), we get for every $t \ge t_0$

11 $$\begin{aligned}&\langle (b+1)\dot{x}(t)+\beta {\nabla }g(x(t))+t(\ddot{x}(t)+\beta {\nabla }^2g(x(t))\dot{x}(t)), b(x(t)-x^*)+t(\dot{x}(t)+\beta {\nabla }g(x(t)))\rangle \nonumber \\ =&\ \langle (b+1-\alpha )\dot{x}(t)+(\beta -t){\nabla }g(x(t))-t\epsilon (t)x(t), b(x(t)-x^*)+t(\dot{x}(t)+\beta {\nabla }g(x(t)))\rangle \nonumber \\ =&\ b(b+1-\alpha )\langle \dot{x}(t),x(t)-x^*\rangle +(b+1-\alpha )t\Vert \dot{x}(t)\Vert ^2+(-t^2+\beta (b+2-\alpha )t\langle \dot{x}(t),{\nabla }g(x(t))\rangle \nonumber \\&+(\beta ^2 t-\beta t^2)\Vert {\nabla }g(x(t))\Vert ^2-\epsilon (t)t^2\langle \dot{x}(t),x(t)\rangle -\beta \epsilon (t)t^2\langle {\nabla }g(x(t)),x(t)\rangle \nonumber \\&-bt\left\langle \left( 1-\frac{\beta }{t}\right) {\nabla }g(x(t))+\epsilon (t)x(t), x(t)-x^*\right\rangle . \end{aligned}$$

Let be $t_0':=\max (\beta ,t_0)$. For all $t \ge t_0'$ the function $g_t : \mathcal {H} \rightarrow \mathbb {R}, g_t(x)= \left( 1-\frac{\beta }{t}\right) g(x)+\frac{\epsilon (t)}{2}\Vert x\Vert ^2,$ is strongly convex, thus, one has

$$\begin{aligned}g_t(y)-g_t(x)\ge \langle {\nabla }g_t(x),y-x\rangle +\frac{\epsilon (t)}{2}\Vert y-x\Vert ^2 \ \forall x,y\in \mathcal {H}.\end{aligned}$$

By taking $x:=x(t)$ and $y:=x^*$ we get for every $t\ge t_0'$

12 $$\begin{aligned} -bt\left\langle \left( 1-\frac{\beta }{t}\right) {\nabla }g(x(t))+\epsilon (t)x(t), x(t)-x^*\right\rangle \le&-bt\left( 1-\frac{\beta }{t}\right) (g(x(t))-\min g) -bt\frac{\epsilon (t)}{2}\Vert x(t)\Vert ^2 \nonumber \\&-bt\frac{\epsilon (t)}{2}\Vert x(t)-x^*\Vert ^2 +bt\frac{\epsilon (t)}{2}\Vert x^*\Vert ^2. \end{aligned}$$

From (10), (11) and (12) it follows that for every $t\ge t_0'$ it holds

13 $$\begin{aligned} \dot{\mathcal {E}_{b}}(t) \le&\ \big ((2-b)t-\beta (2-\alpha )\big )\left( g(x(t))-\min g\right) +bt\frac{\epsilon (t)}{2}\Vert x^*\Vert ^2 \nonumber \\&\ +\left( t^2\frac{\dot{\epsilon }(t)}{2}+(2-b)t\frac{\epsilon (t)}{2}\right) \Vert x(t)\Vert ^2-bt\frac{\epsilon (t)}{2}\Vert x(t)-x^*\Vert ^2\nonumber \\&\ +(b+1-\alpha )t\Vert \dot{x}(t)\Vert ^2+(\beta ^2 t-\beta t^2)\Vert {\nabla }g(x(t))\Vert ^2 -\beta \epsilon (t)t^2\langle {\nabla }g(x(t)),x(t)\rangle . \end{aligned}$$

At this point we treat the situations $\alpha >3$ and $\alpha =3$ separately.

**The case** $\alpha > 3$ and $2<b< \alpha -1$. We will carry out the analysis by addressing the settings provided by the conditions (a) and (b) separately.

*Condition *(a)* holds:* Assuming that condition (a) holds, there exist $a >1$ and $t_1\ge t_0'$ such that

$$\begin{aligned}\dot{\epsilon }(t)\le -\frac{a\beta }{2}\epsilon ^2(t) \ \text{ for } \text{ every } t\ge t_1.\end{aligned}$$

Using that

14 $$\begin{aligned} -\beta \epsilon (t)t^2\langle {\nabla }g(x(t)),x(t)\rangle \le \frac{\beta t^2}{a}\Vert {\nabla }g(x(t))\Vert ^2+\frac{a\beta \epsilon ^2(t)t^2}{4}\Vert x(t)\Vert ^2, \end{aligned}$$

(13) leads to the following estimate

15 $$\begin{aligned} \dot{\mathcal {E}_{b}}(t) \le&\ \big ((2-b)t-\beta (2-\alpha )\big )\left( g(x(t))-\min g\right) +bt\frac{\epsilon (t)}{2}\Vert x^*\Vert ^2 \nonumber \\&\ +\left( t^2\frac{\dot{\epsilon }(t)}{2}+(2-b)t\frac{\epsilon (t)}{2}+\frac{a\beta \epsilon ^2(t)t^2}{4}\right) \Vert x(t)\Vert ^2-bt\frac{\epsilon (t)}{2}\Vert x(t)-x^*\Vert ^2\nonumber \\&\ +(b+1-\alpha )t\Vert \dot{x}(t)\Vert ^2+\left( \beta ^2 t-\beta \left( 1-\frac{1}{a}\right) t^2\right) \Vert {\nabla }g(x(t))\Vert ^2, \end{aligned}$$

which holds for every $t\ge t_1.$

Since $a> 1$ and $b > 2$, we notice that for every $t\ge t_1$ it holds

$$\begin{aligned}t^2\frac{\dot{\epsilon }(t)}{2}+(2-b)t\frac{\epsilon (t)}{2}+\frac{a\beta \epsilon ^2(t)t^2}{4}\le 0.\end{aligned}$$

On the other hand, we have that

$$\begin{aligned}\beta ^2 t-\beta \left( 1-\frac{1}{a}\right) t^2\le -\beta \frac{a-1}{2a}t^2 \text{ for } \text{ every } t\ge \frac{2a\beta }{a-1}\end{aligned}$$

and

$$\begin{aligned}(2-b)t-\beta (2-\alpha )\le 0 \text{ for } \text{ every } t\ge \frac{\beta (\alpha -2)}{b-2}.\end{aligned}$$

We define $t_2:=\max \left( t_1,\frac{2a\beta }{a-1},\frac{\beta (\alpha -2)}{b-2}\right)$. According to (15), it holds for every $t\ge t_2$

16 $$\begin{aligned}&\ \dot{\mathcal {E}_{b}}(t) - \big ((2-b)t-\beta (2-\alpha )\big )\left( g(x(t))-\min g\right) -\left( t^2\frac{\dot{\epsilon }(t)}{2}+(2-b)t\frac{\epsilon (t)}{2}+\frac{a\beta \epsilon ^2(t)t^2}{4}\right) \Vert x(t)\Vert ^2 \nonumber \\&\quad \qquad \ +bt\frac{\epsilon (t)}{2}\Vert x(t)-x^*\Vert ^2+(\alpha -1-b)t\Vert \dot{x}(t)\Vert ^2+\beta \frac{a-1}{2a}t^2\Vert {\nabla }g(x(t))\Vert ^2\nonumber \\&\quad \le \ bt\frac{\epsilon (t)}{2}\Vert x^*\Vert ^2. \end{aligned}$$

*Condition *(b)* holds:* Assuming now that condition (b) holds, there exist $a >0$ and $t_1\ge t_0'$ such that

$$\begin{aligned}\epsilon (t)\le \frac{a}{t} \ \text{ for } \text{ every } t\ge t_1.\end{aligned}$$

Further, the monotonicity of ${\nabla }g$ and the fact that ${\nabla }g(x^*)=0$ implies that

$$\begin{aligned}\langle {\nabla }g(x(t)),x(t)-x^*\rangle \ge 0 \text{ for } \text{ every } t\ge t_0.\end{aligned}$$

Using that

17 $$\begin{aligned} -\beta \epsilon (t)t^2\langle {\nabla }g(x(t)),x(t)\rangle \le -\beta \epsilon (t)t^2\langle {\nabla }g(x(t)),x^*\rangle \le \frac{\beta t^3\epsilon (t)}{2a}\Vert {\nabla }g(x(t))\Vert ^2+\frac{a\beta \epsilon (t)t}{2}\Vert x^*\Vert ^2, \end{aligned}$$

(13) leads to the following estimate

18 $$\begin{aligned} \dot{\mathcal {E}_{b}}(t) \le&\ \big ((2-b)t-\beta (2-\alpha )\big )\left( g(x(t))-\min g\right) +(b+a\beta )t\frac{\epsilon (t)}{2}\Vert x^*\Vert ^2 \nonumber \\&\ +\left( t^2\frac{\dot{\epsilon }(t)}{2}+(2-b)t\frac{\epsilon (t)}{2}\right) \Vert x(t)\Vert ^2-bt\frac{\epsilon (t)}{2}\Vert x(t)-x^*\Vert ^2\nonumber \\&\ +(b+1-\alpha )t\Vert \dot{x}(t)\Vert ^2+\left( \beta ^2 t-\beta t^2+\frac{\beta t^3\epsilon (t)}{2a}\right) \Vert {\nabla }g(x(t))\Vert ^2 \end{aligned}$$

for every $$t\ge t_1$$.

Since $b > 2$, we have that for every $t\ge t_1$ it holds

$$\begin{aligned}t^2\frac{\dot{\epsilon }(t)}{2}+(2-b)t\frac{\epsilon (t)}{2}\le 0.\end{aligned}$$

On the other hand, since

$$\begin{aligned}-\beta t^2+\frac{\beta t^3\epsilon (t)}{2a}\le -\frac{\beta }{2}t^2\end{aligned}$$

holds for every $t\ge t_1$, it follows that

19 $$\begin{aligned} \beta ^2 t-\beta t^2+\frac{\beta t^3\epsilon (t)}{2a}\le -\frac{\beta }{4}t^2 \ \text{ for } \text{ every } t\ge \max (t_1,4\beta ). \end{aligned}$$

We recall that

$$\begin{aligned}(2-b)t-\beta (2-\alpha )\le 0 \text{ for } \text{ every } t\ge \frac{\beta (\alpha -2)}{b-2}. \end{aligned}$$

We define $t_2:=\max \left( t_1,4\beta ,\frac{\beta (\alpha -2)}{b-2}\right) .$ According to (18), it holds for every $t \ge t_2$

20 $$\begin{aligned}&\ \dot{\mathcal {E}_{b}}(t) {-} ((2-b)t-\beta (2-\alpha ))\left( g(x(t)){-}\min g\right) -\left( t^2\frac{\dot{\epsilon }(t)}{2}{+}(2-b)t\frac{\epsilon (t)}{2}\right) \Vert x(t)\Vert ^2 \nonumber \\&\qquad +bt\frac{\epsilon (t)}{2}\Vert x(t)-x^*\Vert ^2+(\alpha -1-b)t\Vert \dot{x}(t)\Vert ^2+\frac{\beta }{4}t^2\Vert {\nabla }g(x(t))\Vert ^2\nonumber \\&\quad \le \ (b+a\beta )t\frac{\epsilon (t)}{2}\Vert x^*\Vert ^2. \end{aligned}$$

From now on we will treat the two cases together. According to (16), in case (a), and to (20), in case (b), we obtain

$$\begin{aligned}\dot{\mathcal {E}_{b}}(t)\le lt\frac{\epsilon (t)}{2}\Vert x^*\Vert ^2 \end{aligned}$$

for every $t\ge t_2$, where $l:=b \text{ and } t_2=\max \left( t_1,\frac{2a\beta }{a-1},\frac{\beta (\alpha -2)}{b-2}\right)$, in case (a), and $l:=b+a\beta \text{ and } t_2=\max \left( t_1,4\beta ,\frac{\beta (\alpha -2)}{b-2}\right)$ in case (b).

By integrating the latter inequality on the interval $[t_2,T]$, where $T \ge t_2$ is arbitrarily chosen, we obtain

$$\begin{aligned}\mathcal {E}_{b}(T)\le \mathcal {E}_{b}(t_2)+\frac{l\Vert x^*\Vert ^2}{2}\int _{t_2}^T t\epsilon (t)dt.\end{aligned}$$

On the other hand,

$$\begin{aligned}\mathcal {E}_{b}(t)\ge (t^2-\beta (b+2-\alpha )t)\left( g(x(T))-\min g\right) \ \forall t \ge t_0, \end{aligned}$$

hence, for every $T\ge \max ( \beta (b+2-\alpha ),t_3)$ we get

$$\begin{aligned}0 \le g(x(T))-\min g \le \frac{\mathcal {E}_{b}(t_2)}{T^2-\beta (b+2-\alpha )T}+\frac{l\Vert x^*\Vert ^2}{2}\frac{1}{T^2-\beta (b+2-\alpha )T}\int _{t_2}^T t\epsilon (t)dt.\end{aligned}$$

Obviously,

$$\begin{aligned}\lim _{T\longrightarrow +\infty }\frac{\mathcal {E}_{b}(t_3)}{T^2-\beta (b+2-\alpha )T}=0. \end{aligned}$$

Further, Lemma A.1 applied to the functions $\varphi (t)=t^2$ and $f(t)=\frac{\epsilon (t)}{t}$ provides

$$\begin{aligned}\lim _{T\longrightarrow +\infty }\frac{1}{T^2}\int _{t_2}^T t^2\frac{\epsilon (t)}{t}dt=0, \end{aligned}$$

hence,

$$\begin{aligned}\lim _{T\longrightarrow +\infty }\frac{1}{T^2-\beta (b+2-\alpha )T}\int _{t_2}^T t\epsilon (t)dt=0 \end{aligned}$$

and, consequently,

$$\begin{aligned}\lim _{T\longrightarrow +\infty }g(x(T))=\min g. \end{aligned}$$

**The case** $\alpha =3$ and $b=2$. In this case the energy functional reads

$$\begin{aligned}\mathcal {E}_{2}(t)=(t^2-\beta t)\left( g(x(t))-\min g\right) +\frac{t^2\epsilon (t)}{2}\Vert x(t)\Vert ^2+\frac{1}{2}\Vert 2(x(t)-x^*)+t(\dot{x}(t)+\beta {\nabla }g(x(t)))\Vert ^2 \end{aligned}$$

for every $t \ge t_0$. We will address again the settings provided by the conditions (a) and (b) separately.

*Condition* (a) *holds:* Relation (15) becomes

$$\begin{aligned} \dot{\mathcal {E}_{2}}(t) \le&\ \beta \left( g(x(t))-\min g\right) +t\epsilon (t)\Vert x^*\Vert ^2+\left( t^2\frac{\dot{\epsilon }(t)}{2}+\frac{a\beta \epsilon ^2(t)t^2}{4}\right) \Vert x(t)\Vert ^2-t\epsilon (t)\Vert x(t)-x^*\Vert ^2\nonumber \\&\ +\left( \beta ^2 t-\beta \left( 1-\frac{1}{a}\right) t^2\right) \Vert {\nabla }g(x(t))\Vert ^2\nonumber \end{aligned}$$

for every $t \ge t_1$. Consequently, for $t_3:= \max \left( t_1,\frac{\beta a}{a-1}\right)$, we have

21 $$\begin{aligned} \dot{\mathcal {E}_{2}}(t)\le \beta \left( g(x(t))-g^*\right) +t\epsilon (t)\Vert x^*\Vert ^2 \end{aligned}$$

for every $t \ge t_3$. After multiplication with $(t-\beta )$, it yields

$$\begin{aligned} t(t-\beta )\dot{\mathcal {E}_{2}}(t)\le \beta t(t-\beta )\left( g(x(t))-g^*\right) +t^2(t-\beta )\epsilon (t)\Vert x^*\Vert ^2\le \beta \mathcal {E}_{2}(t)+t^2(t-\beta )\epsilon (t)\Vert x^*\Vert ^2 \end{aligned}$$

for every $t\ge t_3$. Dividing by $(t-\beta )^2$ we obtain

$$\begin{aligned}\frac{t}{t-\beta }\dot{\mathcal {E}_{2}}(t)\le \frac{\beta }{(t-\beta )^2}\mathcal {E}_{2}(t)+\frac{t^2}{t-\beta }\epsilon (t)\Vert x^*\Vert ^2 \end{aligned}$$

or, equivalently,

22 $$\begin{aligned} \frac{d}{dt}\left( \frac{t}{t-\beta }\mathcal {E}_{2}(t)\right) \le \frac{t^2}{t-\beta }\epsilon (t)\Vert x^*\Vert ^2 \text{ for } \text{ every } t\ge t_3. \end{aligned}$$

*Condition* (b) *holds:* We define $t_3:=\max \left( t_1,4\beta \right)$. Relation (18) becomes

23 $$\begin{aligned} \dot{\mathcal {E}_{2}}(t)\le \beta \left( g(x(t))-g^*\right) +\frac{2+a\beta }{2}t\epsilon (t)\Vert x^*\Vert ^2, \end{aligned}$$

for every $t\ge t_3$. Repeating the above steps for the inequality (23) we obtain

24 $$\begin{aligned} \frac{d}{dt}\left( \frac{t}{t-\beta }\mathcal {E}_{2}(t)\right) \le \frac{2+a_1\beta }{2}\frac{t^2}{t-\beta }\epsilon (t)\Vert x^*\Vert ^2 \text{ for } \text{ every } t\ge t_3. \end{aligned}$$

From now on we will treat the two cases together. According to (22), in case (a), and to (24), in case (b), we obtain

$$\begin{aligned}\frac{d}{dt}\left( \frac{t}{t-\beta }\mathcal {E}_{2}(t)\right) \le l\frac{t^2}{t-\beta }\epsilon (t)\Vert x^*\Vert ^2\end{aligned}$$

for every $t\ge t_3$, where $l:=1 \text{ and } t_3=\max \left( t_1,\frac{\beta (\alpha -1)}{b-2}\right)$, in case (a), and $l:=\frac{2+a\beta }{2} \text{ and } t_3=\max (t_1,4\beta )$ in case (b).

By integrating the latter inequality on an interval $[t_3,T]$, where $T \ge t_3$ is arbitrarily chosen, we obtain

$$\begin{aligned}\frac{T}{T-\beta }\mathcal {E}_{2}(T)\le \frac{t_3}{t_3-\beta }\mathcal {E}_{2}(t_3)+l\Vert x^*\Vert ^2\int _{t_3}^T \frac{t^2}{t-\beta }\epsilon (t) dt.\end{aligned}$$

On the other hand,

$$\begin{aligned}\mathcal {E}_{2}(t)\ge (t^2-\beta t)\left( g(x(t))-\min g\right) \end{aligned}$$

for every $t \ge t_0$, hence, for every $T \ge \max ( \beta ,t_3)=t_3$ we get

$$\begin{aligned}0 \le g(x(T))-\min g \le \frac{1}{T^2} \frac{t_3}{t_3-\beta }\mathcal {E}_{2}(t_3)+l\Vert x^*\Vert ^2\frac{1}{T^2}\int _{t_3}^T \frac{t^2}{t-\beta }\epsilon (t)dt.\end{aligned}$$

Obviously,

$$\begin{aligned}\lim _{T\longrightarrow +\infty }\frac{1}{T^2} \frac{t_3}{t_3-\beta }\mathcal {E}_{2}(t_3)=0.\end{aligned}$$

Lemma A.1, applied this time to the functions $\varphi (t)=\frac{t^3}{t-\beta }$ and $f(t)=\frac{\epsilon (t)}{t}$, yields

$$\begin{aligned}\lim _{T\longrightarrow +\infty } \frac{T-\beta }{T^3}\int _{t_3}^T \frac{t^3}{t-\beta }\frac{\epsilon (t)}{t}dt=0.\end{aligned}$$

Consequently,

$$\begin{aligned}\lim _{T\longrightarrow +\infty }\frac{1}{T^2}\int _{t_3}^T \frac{t^2}{t-\beta }\epsilon (t)dt=0,\end{aligned}$$

hence

$$\begin{aligned}\lim _{T\longrightarrow +\infty }g(x(T))=\min g.\end{aligned}$$

$$\square $$

### Remark 3.2

One can easily notice that, in case $\beta >0$, the fact that there exist $a >1$ and $t_1 \ge t_0$ such that $\dot{\epsilon }(t)\le -\frac{a\beta }{2}\epsilon ^2(t)$ for every $t\ge t_1$ implies that $\int _{t_0}^{+\infty }\frac{\epsilon (t)}{t}dt<+\infty$.

The next theorem shows that, by strengthening the integrability condition $\int _{t_0}^{+\infty }\frac{\epsilon (t)}{t}dt<+\infty$ (which is actually required in both settings (a) and (b) of Theorem 3.1), a rate of $\mathcal {O}(1/t^2)$ ca be guaranteed for the convergence of *g*(*x*(*t*)) to $\min g$.

### Theorem 3.3

Let *x* be the unique global $C^2$-solution of (5). Assume that

$$\begin{aligned}\int _{t_0}^{+\infty }t\epsilon (t)dt<+\infty \end{aligned}$$

and that one of the following conditions is fulfilled:

- there exist $a>1$ and $t_1\ge t_0$ such that
  $$\begin{aligned}\dot{\epsilon }(t)\le -\frac{a\beta }{2}\epsilon ^2(t) \text{ for } \text{ every } t\ge t_1;\end{aligned}$$
- there exist $a>0$ and $t_1\ge t_0$ such that
  $$\begin{aligned}\epsilon (t)\le \frac{a}{t} \text{ for } \text{ every } t\ge t_1.\end{aligned}$$

If $$\alpha \ge 3$$, then

$$\begin{aligned}g(x(t))-\min g = \mathcal {O}\left( \frac{1}{t^2}\right) .\end{aligned}$$

In addition, if $\alpha >3$, then the trajectory *x* is bounded and

$$\begin{aligned}t\left( g(x(t))-\min g\right) ,\,t\Vert \dot{x}(t)\Vert ^2,\,t\epsilon (t)\Vert x(t)-x^*\Vert ^2,\,t\epsilon (t)\Vert x(t)\Vert ^2,\,t^2\Vert {\nabla }g(x(t))\Vert ^2\in L^1([t_0,+\infty ),\mathbb {R})\end{aligned}$$

for every arbitrary $x^* \in \hbox {argmin}g$.

### Proof

Let be $x^* \hbox {argmin}g$ and $2 \le b \le \alpha -1$ fixed. We will use the energy functional introduced in the proof of the previous theorem and some of the estimate we derived for it. We will treat again the situations $\alpha >3$ and $\alpha =3$ separately.

**The case** $\alpha > 3$ and $2<b< \alpha -1$. As we already noticed in the proof of Theorem 3.1, according to (16), in case (a), and to (20), in case (b), we have

$$\begin{aligned}\dot{\mathcal {E}_{b}}(t)\le lt\frac{\epsilon (t)}{2}\Vert x^*\Vert ^2 \ \text{ for } \text{ every } \ t \ge t_2,\end{aligned}$$

where $l:=b \text{ and } t_2=\max \left( t_1,\frac{2a\beta }{a-1},\frac{\beta (\alpha -2)}{b-2}\right)$, in case (a), and $l:=b+a\beta \text{ and } t_2=\max \left( t_1,4\beta ,\frac{\beta (\alpha -2)}{b-2}\right)$ in case (b).

Using that $t\epsilon (t)\in L^1([t_0,+\infty ),\mathbb {R})$ and that $t \mapsto \mathcal {E}_{b}(t)$ is bounded from below, from Lemma A.2 it follows that the limit $\lim _{t\longrightarrow +\infty }\mathcal {E}_{b}(t)$ exists. Consequently, $t \mapsto \mathcal {E}_{b}(t)$ is bounded, which implies that there exist $K>0$ and $t'\ge t_0$ such that

$$\begin{aligned}0 \le g(x(t))-\min g\le \frac{K}{t^2} \text{ for } \text{ every } t\ge t'. \end{aligned}$$

In addition, the function $t \mapsto \Vert x(t)-x^*\Vert ^2$ is bounded, hence the trajectory *x* is bounded. Since $t \mapsto \Vert b(x(t)-x^*)+t(\dot{x}(t)+\beta {\nabla }g(x(t)))\Vert ^2$ is also bounded, the inequality

$$\begin{aligned}\Vert t(\dot{x}(t)+\beta {\nabla }g(x(t)))\Vert ^2 \le 2\Vert b(x(t)-x^*)+t(\dot{x}(t)+\beta {\nabla }g(x(t)))\Vert ^2+2b^2\Vert x(t)-x^*\Vert ^2,\end{aligned}$$

which is true for every $t \ge t_0$, leads to

$$\begin{aligned}\Vert \dot{x}(t)+\beta {\nabla }g(x(t))\Vert = \mathcal {O}\left( \frac{1}{t}\right) .\end{aligned}$$

By integrating relation (16), in case (a), and relation (20), in case (b), on an interval $[t_2, s]$, where $s \ge t_3$ is arbitrarily chosen, and by letting afterwards *s* converge to $+\infty$, we obtain

$$\begin{aligned}t\left( g(x(t)){-}\min g \right) ,\,t\Vert \dot{x}(t)\Vert ^2,\,t\epsilon (t)\Vert x(t){-}x^*\Vert ^2, t^2\Vert {\nabla }g(x(t))\Vert ^2 \in L^1([t_0,+\infty ),\mathbb {R}).\end{aligned}$$

The boundedness of the trajectory and the condition on the Tikhonov parametrization guarantee that

$$\begin{aligned}t\epsilon (t)\Vert x(t)\Vert ^2\in L^1([t_0,+\infty ),\mathbb {R}).\end{aligned}$$

**The case** $\alpha = 3$ and $b=2$. As we already noticed in the proof of Theorem 3.1, according to (22), in case (a), and to (24), in case (b), we obtain

$$\begin{aligned} \frac{d}{dt}\left( \frac{t}{t-\beta }\mathcal {E}_{2}(t)\right) \le l\frac{t^2}{t-\beta }\epsilon (t)\Vert x^*\Vert ^2 \ \text{ for } \text{ every } \ t \ge t_3, \end{aligned}$$

where $l=1 \text{ and } t_3=\max \left( t_1,\frac{\beta (\alpha -1)}{b-2}\right)$, in case (a), and $l=\frac{2+a\beta }{2} \text{ and } t_3=\max (t_1,4\beta )$ in case (b).

Since $t\epsilon (t)\in L^1([t_0,+\infty ),\mathbb {R})$ and $\epsilon (t)$ is nonnegative, obviously $\frac{t^2}{t-\beta }\epsilon (t)\Vert x^*\Vert ^2\in L^1([t_2,+\infty ),\mathbb {R})$. Using that $t \mapsto \frac{t}{t-\beta }\mathcal {E}_{2}(t)$ is bounded from below, from Lemma A.2 it follows that the limit $\lim _{t\longrightarrow +\infty }\frac{t}{t-\beta }\mathcal {E}_{2}(t)$ exists. Consequently, the limit $\lim _{t\longrightarrow +\infty }\mathcal {E}_{2}(t)$ also exists and $t \mapsto \mathcal {E}_{2}(t)$ is bounded. This implies that there exist $K>0$ and $t'\ge t_0$ such that

$$\begin{aligned}0 \le g(x(t))-\min g\le \frac{K}{t^2} \text{ for } \text{ every } t\ge t'.\end{aligned}$$

$$\square $$

The next result shows that the statements of Theorem 3.3 can be strengthened in case $\alpha >3$.

### Theorem 3.4

Let *x* be the unique global $C^2$-solution of (5). Assume that

$$\begin{aligned}\int _{t_0}^{+\infty }t\epsilon (t)dt<+\infty \end{aligned}$$

and that one of the following conditions is fulfilled:

- there exist $a>1$ and $t_1\ge t_0$ such that
  $$\begin{aligned}\dot{\epsilon }(t)\le -\frac{a\beta }{2}\epsilon ^2(t) \text{ for } \text{ every } t\ge t_1;\end{aligned}$$
- there exist $a>0$ and $t_1\ge t_0$ such that
  $$\begin{aligned}\epsilon (t)\le \frac{a}{t} \text{ for } \text{ every } t\ge t_1.\end{aligned}$$

Let be an arbitrary $x^* \in \hbox {argmin}g$. If $\alpha > 3$, then

$$\begin{aligned}t\langle {\nabla }g(x(t)),x(t)-x^*\rangle \in L^1([t_0,+\infty ),\mathbb {R})\end{aligned}$$

and the limits

$$\begin{aligned}\lim _{t\longrightarrow +\infty }\Vert x(t)-x^*\Vert \in \mathbb {R}\ \text{ and } \ \lim _{t\longrightarrow +\infty }t\langle \dot{x}(t)+\beta {\nabla }g(x(t)),x(t)-x^*\rangle \in \mathbb {R}\end{aligned}$$

exist. In addition,

$$\begin{aligned}g(x(t))-\min g=o\left( \frac{1}{t^2}\right) ,\,\Vert \dot{x}(t)+\beta {\nabla }g(x(t))\Vert =o\left( \frac{1}{t}\right) \ \text{ and } \ \lim _{t\longrightarrow +\infty }t^2\epsilon (t)\Vert x(t)\Vert ^2=0.\end{aligned}$$

### Proof

Since $\alpha >3$ we can choose $2<b< \alpha -1$. From (10) and (11) we have that

25 $$\begin{aligned} \dot{\mathcal {E}_{b}}(t) =&\ (2t-\beta (b+2-\alpha ))\left( g(x(t))-\min g\right) +\left( t^2\frac{\dot{\epsilon }(t)}{2}+t\epsilon (t)\right) \Vert x(t)\Vert ^2\nonumber \\&\ {+}(b+1-\alpha )t\Vert \dot{x}(t)\Vert ^2{+}(\beta ^2 t-\beta t^2)\Vert {\nabla }g(x(t))\Vert ^2{-}\beta \epsilon (t)t^2\langle {\nabla }g(x(t)),x(t)\rangle \nonumber \\&\ -bt\left\langle \left( 1-\frac{\beta }{t}\right) {\nabla }g(x(t)){+}\epsilon (t)x(t), x(t)-x^*\right\rangle \text{ for } \text{ every } t\ge t_0. \end{aligned}$$

We will address the settings provided by the conditions (a) and (b) separately.

*Condition* (a) *holds:* In this case we estimate $-\beta \epsilon (t)t^2\langle {\nabla }g(x(t)),x(t)\rangle$ just as in (14) and from (25) we obtain

26 $$\begin{aligned} \dot{\mathcal {E}_{b}}(t) \le&\ (2t-\beta (b+2-\alpha ))\left( g(x(t))-\min g\right) +\left( t^2\frac{\dot{\epsilon }(t)}{2}+t\epsilon (t)+\frac{a\beta \epsilon ^2(t)t^2}{4}\right) \Vert x(t)\Vert ^2 \nonumber \\&\ +(b+1-\alpha )t\Vert \dot{x}(t)\Vert ^2+\left( \beta ^2 t-\beta \left( 1-\frac{1}{a}\right) t^2\right) \Vert {\nabla }g(x(t))\Vert ^2\nonumber \\&\ -bt\left\langle \left( 1-\frac{\beta }{t}\right) {\nabla }g(x(t))+\epsilon (t)x(t), x(t)-x^*\right\rangle \text{ for } \text{ every } t\ge t_0. \end{aligned}$$

We define $t_2:=\max \left( \beta ,t_1,\frac{\beta a}{a-1}\right) .$ By using condition (a), neglecting the nonpositive terms and afterwards integrating on the interval $[t_2,t]$, with arbitrary $t \ge t_2$, we obtain

27 $$\begin{aligned} \int _{t_2}^t bs\left\langle \left( 1-\frac{\beta }{s}\right) {\nabla }g(x(s)), x(s)-x^*\right\rangle \le&\ \mathcal {E}_{b}(t_2)-\mathcal {E}_{b}(t)+\int _{t_2}^t (2s-\beta (b+2-\alpha ))\left( g(x(s))-\min g\right) ds \nonumber \\&-\int _{t_2}^t bs\left( 1-\frac{\beta }{s}\right) \left\langle \epsilon (s)x(s), x(s)-x^*\right\rangle +\int _{t_2}^t s\epsilon (s)\Vert x(s)\Vert ^2ds. \end{aligned}$$

For every $s\ge t_2$, by the monotonicity of ${\nabla }g$, we have $\left\langle {\nabla }g(x(s)), x(s)-x^*\right\rangle \ge 0$. Further, it holds

$$\begin{aligned}bs\left( 1-\frac{\beta }{s}\right) \epsilon (s)\left| \left\langle x(s), x(s)-x^*\right\rangle \right| \le \left( 1-\frac{\beta }{s}\right) \frac{bs\epsilon (s)}{2}(\Vert x(s)\Vert ^2+\Vert x(s)-x^*\Vert ^2).\end{aligned}$$

By letting in (27) *s* converge to $+\infty$ and by taking into account that, according to Theorem 3.3,

$$\begin{aligned}t\epsilon (t)\Vert x(t)\Vert ^2,\,t\epsilon (t)\Vert x(t)-x^*\Vert ^2,\,(2t-\beta (b+2-\alpha ))\left( g(x(t))-g^*\right) \in L^1([t_0,+\infty ),\mathbb {R})\end{aligned}$$

28 $$\begin{aligned} t\langle {\nabla }g(x(t)),x(t)-x^*\rangle \in L^1([t_0,+\infty ),\mathbb {R}). \end{aligned}$$

*Condition* (b) *holds:* In this case we estimate $-\beta \epsilon (t)t^2\langle {\nabla }g(x(t)),x(t)\rangle$ just as in (17) and from (25) we obtain

29 $$\begin{aligned} \dot{\mathcal {E}_{b}}(t) \le&\ (2t-\beta (b+2-\alpha ))\left( g(x(t))-\min g\right) +\left( t^2\frac{\dot{\epsilon }(t)}{2}+t\epsilon (t)\right) \Vert x(t)\Vert ^2\nonumber \\&\ +(b+1-\alpha )t\Vert \dot{x}(t)\Vert ^2+\left( \beta ^2 t-\beta t^2+\frac{\beta \epsilon (t)t^3}{2a}\right) \Vert {\nabla }g(x(t))\Vert ^2+\frac{a_1\beta \epsilon (t)t}{2}\Vert x^*\Vert ^2\nonumber \\&\ -bt\left\langle \left( 1-\frac{\beta }{t}\right) {\nabla }g(x(t))+\epsilon (t)x(t), x(t)-x^*\right\rangle \text{ for } \text{ every } t\ge t_0. \end{aligned}$$

We define $t_2:=\max \left( 4\beta ,t_1\right) .$ According to (19) we have that $\beta ^2 t-\beta t^2+\frac{\beta \epsilon (t)t^3}{2a_1}\le 0$ for every $t\ge t_2$. By using condition (b), neglecting the nonpositive terms and afterwards integrating on the interval $[t_2,t]$, with arbitrary $t \ge t_2$, we obtain

30 $$\begin{aligned} \int _{t_2}^t bs\left\langle \left( 1-\frac{\beta }{s}\right) {\nabla }g(x(s)), x(s)-x^*\right\rangle \le&\ \mathcal {E}_{b}(t_2)-\mathcal {E}_{b}(t)+\int _{t_2}^t (2s-\beta (b+2-\alpha ))\left( g(x(s))-\min g\right) ds \nonumber \\&\ -\int _{t_2}^t bs\left( 1-\frac{\beta }{s}\right) \left\langle \epsilon (s)x(s), x(s)-x^*\right\rangle +\int _{t_2}^t s\epsilon (s)\Vert x(s)\Vert ^2ds \nonumber \\&\ +\frac{a\beta }{2}\Vert x^*\Vert ^2\int _{t_2}^t s\epsilon (s)ds. \end{aligned}$$

From here, by using the similar arguments as for the case (a), we obtain (28).

Consider now, $b_1,b_2\in (2, \alpha -1),\,b_1\ne b_2.$ Then for every $t \ge t_0$ we have

$$\begin{aligned}&\mathcal {E}_{b_1}(t)-\mathcal {E}_{b_2}(t)=(b_1-b_2)\left( -\beta t(g(x(t))-\min g)+t\langle \dot{x}(t)+\beta {\nabla }g(x(t)),x(t)-x^*\rangle \right. \\&\left. \quad +\,\frac{\alpha -1}{2}\Vert x(t)-x^*\Vert ^2\right) . \end{aligned}$$

According to Theorem 3.3, the limits

$$\begin{aligned} \lim _{t\longrightarrow +\infty }(\mathcal {E}_{b_1}(t)-\mathcal {E}_{b_2}(t)) \in \mathbb {R}\ \text{ and } \ \lim _{t\longrightarrow +\infty }t(g(x(t))-g^*) \in \mathbb {R}\end{aligned}$$

exist, consequently, the limit

$$\begin{aligned}\lim _{t\longrightarrow +\infty }\left( t\langle \dot{x}(t)+\beta {\nabla }g(x(t)),x(t)-x^*\rangle +\frac{\alpha -1}{2}\Vert x(t)-x^*\Vert ^2\right) \end{aligned}$$

also exists. For every $t \ge t_0$ we define

$$\begin{aligned}k(t)=t\langle \dot{x}(t)+\beta {\nabla }g(x(t)),x(t)-x^*\rangle +\frac{\alpha -1}{2}\Vert x(t)-x^*\Vert ^2\end{aligned}$$

and

$$\begin{aligned}q(t)=\frac{1}{2}\Vert x(t)-x^*\Vert ^2+\beta \int _{t_0}^t\langle {\nabla }g(x(s)),x(s)-x^*\rangle ds.\end{aligned}$$

Then

$$\begin{aligned}(\alpha -1)q(t)+t\dot{q}(t)=k(t)+\beta (\alpha -1)\int _{t_0}^t\langle {\nabla }g(x(s)),x(s)-x^*\rangle ds \ \text{ for } \text{ every } \ t \ge t_0.\end{aligned}$$

From (28) and the fact that *k*(*t*) has a limit whenever $t\longrightarrow +\infty$, we obtain that $(\alpha -1)q(t)+t\dot{q}(t)$ has a limit when $t\longrightarrow +\infty$. According to Lemma 4.6, *q*(*t*) has a limit when $t\longrightarrow +\infty .$ By using (28) again we obtain that the limit

$$\begin{aligned}\lim _{t\longrightarrow +\infty }\Vert x(t)-x^*\Vert \in \mathbb {R}\end{aligned}$$

exists and, consequently, the limit

$$\begin{aligned}\lim _{t\longrightarrow +\infty }t\langle \dot{x}(t)+\beta {\nabla }g(x(t)),x(t)-x^*\rangle \in \mathbb {R}\end{aligned}$$

also exists. On the other hand, we notice that for every $t \ge t_0$ the energy functional can be written as

31 $$\begin{aligned} \mathcal {E}_{b}(t)=&\ (t^2-\beta (b+2-\alpha )t)\left( g(x(t))-\min g\right) +\frac{t^2\epsilon (t)}{2}\Vert x(t)\Vert ^2 \nonumber \\&\ +\frac{t^2}{2}\Vert \dot{x}(t)+\beta {\nabla }g(x(t))\Vert ^2+bt\langle \dot{x}(t)+\beta {\nabla }g(x(t)),x(t)-x^*\rangle +\frac{b(\alpha -1)}{2}\Vert x(t)-x^*\Vert ^2. \end{aligned}$$

Since the limits

$$\begin{aligned}\lim _{t\longrightarrow +\infty }\mathcal {E}_{b}(t) \in \mathbb {R}\ \text{ and } \ \lim _{t\longrightarrow +\infty }\left( bt\langle \dot{x}(t)+\beta {\nabla }g(x(t)),x(t)-x^*\rangle +\frac{b(\alpha -1)}{2}\Vert x(t)-x^*\Vert ^2\right) \in \mathbb {R}\end{aligned}$$

exist, it follows that the limit

$$\begin{aligned}\lim _{t\longrightarrow +\infty }\left( (t^2-\beta (b+2-\alpha )t)\left( g(x(t))-\min g\right) +\frac{t^2\epsilon (t)}{2}\Vert x(t)\Vert ^2+\frac{t^2}{2}\Vert \dot{x}(t)+\beta {\nabla }g(x(t))\Vert ^2\right) \in \mathbb {R}\end{aligned}$$

exists, too.

We define

$$\begin{aligned}\varphi :[t_0,+\infty )\longrightarrow \mathbb {R}, \ \varphi (t){=}(t^2{-}\beta (b{+}2{-}\alpha )t)\left( g(x(t)){-}g^*\right) {+}\frac{t^2\epsilon (t)}{2}\Vert x(t)\Vert ^2{+}\frac{t^2}{2}\Vert \dot{x}(t){+}\beta {\nabla }g(x(t))\Vert ^2,\end{aligned}$$

and notice that for sufficiently large *t* it holds

$$\begin{aligned}0\le \frac{\varphi (t)}{t}\le 2t\left( g(x(t))-\min g\right) +\frac{t\epsilon (t)}{2}\Vert x(t)\Vert ^2+\frac{t}{2}\Vert \dot{x}(t)+\beta {\nabla }g(x(t))\Vert ^2.\end{aligned}$$

According to Theorem 3.3 the right hand side of the above inequality is of class $L^1([t_0,+\infty ),\mathbb {R}).$

Hence,

$$\begin{aligned}\frac{\varphi (t)}{t}\in L^1([t_0,+\infty ),\mathbb {R}).\end{aligned}$$

Since $\frac{1}{t}\not \in L^1([t_0,+\infty ),\mathbb {R})$ and the limit $\lim _{t \longrightarrow +\infty } \varphi (t) \in \mathbb {R}$ exists, it must hold that $\lim _{t\longrightarrow +\infty }\varphi (t)=0.$ Consequently,

$$\begin{aligned}\lim _{t\longrightarrow +\infty }(t^2-\beta (b+2-\alpha )t)\left( g(x(t))-\min g\right) =\lim _{t\longrightarrow +\infty }\frac{t^2\epsilon (t)}{2}\Vert x(t)\Vert ^2=\lim _{t\longrightarrow +\infty }\frac{t^2}{2}\Vert \dot{x}(t)+\beta {\nabla }g(x(t))\Vert ^2=0\end{aligned}$$

and the proof is complete. $\square$

Working in the hypotheses of Theorem 3.4 we can prove also the weak convergence of the trajectories generated by (5) to a minimizer of the objective function *g*.

### Theorem 3.5

Let *x* be the unique global $C^2$-solution of (5). Assume that

$$\begin{aligned}\int _{t_0}^{+\infty }t\epsilon (t)dt<+\infty \end{aligned}$$

and that one of the following conditions is fulfilled:

- there exist $a>1$ and $t_1\ge t_0$ such that
  $$\begin{aligned}\dot{\epsilon }(t)\le -\frac{a\beta }{2}\epsilon ^2(t) \text{ for } \text{ every } t\ge t_1;\end{aligned}$$
- there exist $a>0$ and $t_1\ge t_0$ such that
  $$\begin{aligned}\epsilon (t)\le \frac{a}{t} \text{ for } \text{ every } t\ge t_1.\end{aligned}$$

If $\alpha > 3$, then *x*(*t*) converges weakly to an element in $\hbox {argmin}g$ as $t \longrightarrow +\infty$.

### Proof

We will to apply the continuous version of the Opial Lemma (Lemma A.3) for $S=\hbox {argmin}g.$ According to Theorem 3.4, the limit

$$\begin{aligned}\lim _{t\longrightarrow +\infty }\Vert x(t)-x^*\Vert \in \mathbb {R}\end{aligned}$$

exists for every $x^*\in \hbox {argmin}g$.

Further, let $\overline{x}\in \mathcal {H}$ be a weak sequential limit point of *x*(*t*). This means that there exists a sequence $(t_n)_{n \in \mathbb {N}} \subseteq [t_0, +\infty )$ such that $\lim _{n \longrightarrow \infty } t_n = +\infty$ and $x(t_n)$ converges weakly to $\overline{x}$ as $n \longrightarrow \infty$. Since *g* is weakly lower semicontinuous, we have that

$$\begin{aligned}g(\overline{x})\le \liminf _{n\longrightarrow +\infty }g(x(t_n)).\end{aligned}$$

On the other hand, according to Theorem 3.3,

$$\begin{aligned}\lim _{t\longrightarrow +\infty }g(x(t))=\min g,\end{aligned}$$

consequently one has $g(\overline{x})\le \min g$, which shows that $\overline{x}\in \hbox {argmin}g.$

The convergence of the trajectory is a consequence of Lemma A.3. $\square$

### Remark 3.6

We proved in this section that the convergence rate of $o\left( \frac{1}{t^2}\right)$ for *g*(*x*(*t*)), the converge rate of $o\left( \frac{1}{t}\right)$ for $\Vert \dot{x}(t)+\beta {\nabla }g(x(t))\Vert$ and the weak convergence of the trajectory to a minimizer of *g* that have been obtained in [11] for the dynamical system with Hessian driven damping (3) are preserved when this system is enhanced with a Tikhonov regularization term. In addition, in the case when the Hessian driven damping term is removed, which is the case when $\beta =0$, we recover the results provided in [8] for the dynamical system (4) with Tikhonov regularization term. In this setting, we have to assume in Theorem 3.1 just that $\int _{t_0}^{+\infty }\frac{\epsilon (t)}{t}dt<+\infty$, and in the theorems 3.3 - 3.5 just that $\int _{t_0}^{+\infty }t\epsilon (t)dt<+\infty$, since condition (a) is automatically fulfilled.

## Strong convergence to the minimum norm solution

In this section we will continue the investigations we did at the end of Section 3, by working in the same setting, on the behaviour of the trajectory of the dynamical system (5) by concentrating on strong convergence. In particular, we will provide conditions on the Tikhonov parametrization $t \mapsto \epsilon (t)$ which will guarantee that the trajectory converges to a minimum norm solution of *g*, which is the element of minimum norm of the nonempty convex closed set $\hbox {argmin}g$. We start with the following result.

### Lemma 4.1

Let *x* be the unique global $C^2$-solution of (5). For $x^*\in \hbox {argmin}g$ we introduce the function

$$\begin{aligned}h_{x^*}:[t_0,+\infty )\longrightarrow \mathbb {R}\ h_{x^*}(t)=\frac{1}{2}\Vert x(t)-x^*\Vert ^2.\end{aligned}$$

If $\alpha > 0$ and $\beta \ge 0$, then

$$\begin{aligned}\sup _{t\ge t_0}\Vert \dot{x}(t)\Vert <+\infty \ \text{ and } \ \frac{1}{t}\Vert \dot{x}(t)\Vert ^2\in L^1([t_0,+\infty ),\mathbb {R}).\end{aligned}$$

In addition,

$$\begin{aligned}\sup _{t\ge t_0}\frac{1}{t}|\dot{h}_{x^*}(t)|<+\infty .\end{aligned}$$

### Proof

We consider the following energy functional

32 $$\begin{aligned} W: [t_0,+\infty ) \rightarrow \mathbb {R}, \ W(t)=g(x(t))+\frac{1}{2}\Vert \dot{x}(t)\Vert ^2+\frac{\epsilon (t)}{2}\Vert x(t)\Vert ^2. \end{aligned}$$

By using (5) we have for every $t \ge t_0$

$$\begin{aligned} \dot{W}(t)=&\ \langle {\nabla }g(x(t),\dot{x}(t)\rangle +\langle \ddot{x}(t),\dot{x}(t)\rangle +\frac{\dot{\epsilon }(t)}{2}\Vert x(t)\Vert ^2+\epsilon (t)\langle \dot{x}(t),x(t)\rangle \\ =&\ \langle {\nabla }g(x(t),\dot{x}(t)\rangle +\frac{\dot{\epsilon }(t)}{2}\Vert x(t)\Vert ^2+\epsilon (t)\langle \dot{x}(t),x(t)\rangle \\&\ +\left\langle -\frac{\alpha }{t}\dot{x}(t)-\beta {\nabla }^2g(x(t))\dot{x}(t)-{\nabla }g(x(t))-\epsilon (t)x(t),\dot{x}(t)\right\rangle \\ =&\ -\frac{\alpha }{t}\Vert \dot{x}(t)\Vert ^2+\frac{\dot{\epsilon }(t)}{2}\Vert x(t)\Vert ^2-\beta \langle {\nabla }^2 g(x(t))\dot{x}(t),\dot{x}(t)\rangle . \end{aligned}$$

From here, invoking the convexity of *g*, it follows

33 $$\begin{aligned} \dot{W}(t)\le -\frac{\alpha }{t}\Vert \dot{x}(t)\Vert ^2+\frac{\dot{\epsilon }(t)}{2}\Vert x(t)\Vert ^2, \end{aligned}$$

for every $t \ge t_0$. Since $\epsilon$ is nonincreasing this leads further to

34 $$\begin{aligned} \dot{W}(t)\le -\frac{\alpha }{t}\Vert \dot{x}(t)\Vert ^2 \text{ for } \text{ every } t\ge t_0, \end{aligned}$$

therefore the energy *W* is nonincreasing. Since *W* is bounded from bellow, there exists $\lim _{t\longrightarrow +\infty }W(t)\in \mathbb {R}.$ Consequently, $t \mapsto W(t)$ is bounded on $[t_0,+\infty )$ from which, since *g* is bounded from bellow, we obtain that

$$\begin{aligned}\sup _{t\ge t_0}\Vert \dot{x}(t)\Vert =K<+\infty .\end{aligned}$$

By integrating (34) on an interval $[t_0,t]$ for arbitrary $t > t_0$ it yields

$$\begin{aligned}\int _{t_0}^t \frac{\alpha }{s}\Vert \dot{x}(s)\Vert ^2 ds\le W(t_0)-W(t),\end{aligned}$$

which, by letting $t\longrightarrow +\infty$, leads to

$$\begin{aligned}\frac{1}{t}\Vert \dot{x}(t)\Vert ^2\in L^1([t_0,+\infty ),\mathbb {R}).\end{aligned}$$

Further, for every $t \ge t_0$ we have that

$$\begin{aligned}|\dot{h}_{x^*}(t)|=|\langle \dot{x}(t),x(t)-x^*\rangle |\le \Vert \dot{x}(t)\Vert \Vert x(t)-x^*\Vert \end{aligned}$$

and

$$\begin{aligned}\Vert |x(t)-x^*\Vert \le \Vert x(t)-x(t_0)\Vert +\Vert x(t_0)-x^*\Vert \le \sup _{t\ge t_0}\Vert \dot{x}(t)\Vert (t-t_0)+\Vert x(t_0)-x^*\Vert ,\end{aligned}$$

hence,

$$\begin{aligned} \frac{1}{t}|\dot{h}_{x^*}(t)|\le&\sup _{t\ge t_0}\Vert \dot{x}(t)\Vert \left( \sup _{t\ge t_0}\Vert \dot{x}(t)\Vert \left( 1-\frac{t_0}{t}\right) +\frac{1}{t}\Vert x(t_0)-x^*\Vert \right) \\ \le&\sup _{t\ge t_0}\Vert \dot{x}(t)\Vert \left( \sup _{t\ge t_0}\Vert \dot{x}(t)\Vert +\frac{1}{t_0}\Vert x(t_0)-x^*\Vert \right) \in \mathbb {R}. \end{aligned}$$

$$\square $$

For each $\epsilon > 0,$ we denote by $x_{\epsilon }$ the unique solution of the strongly convex minimization problem

$$\begin{aligned}x_{\epsilon } = \mathop {\hbox {argmin}}\limits _{x\in \mathcal {H}}\left( g(x)+\frac{\epsilon }{2}\Vert x\Vert ^2\right) .\end{aligned}$$

In virtue of the Fermat rule, this is equivalent to

$$\begin{aligned}{\nabla }g(x_\epsilon )+\epsilon x_\epsilon =0.\end{aligned}$$

It is well known that the Tikhonov approximation curve $\epsilon \longrightarrow x_\epsilon$ satisfies $\lim _{\epsilon \longrightarrow 0}x_\epsilon =x^*$, where $x^*= \hbox {argmin}\{\Vert x\Vert : x \in \hbox {argmin}g\}$ is the element of minimum norm of the nonempty convex closed set $\hbox {argmin}g$. Since ${\nabla }g$ is monotone, for every $\epsilon >0$ it holds $\langle {\nabla }g(x_\epsilon )-{\nabla }g(x^*), x_\epsilon -x^*\rangle \ge 0$, that is $\langle -\epsilon x_\epsilon ,x_\epsilon -x^*\rangle \ge 0$. Hence,$-\Vert x_\epsilon \Vert ^2+\langle x_\epsilon ,x^*\rangle \ge 0$, which, by using the Cauchy-Schwarz inequality, implies

$$\begin{aligned}\Vert x_\epsilon \Vert \le \Vert x^*\Vert \ \text{ for } \text{ every } \ \epsilon >0.\end{aligned}$$

### Strong ergodic convergence

We will start by proving a strong ergodic convergence result for the trajectory of (5).

#### Theorem 4.2

Let *x* be the unique global $C^2$-solution of (5). Assume that

$$\begin{aligned}\int _{t_0}^{+\infty }\frac{\epsilon (t)}{t}dt=+\infty .\end{aligned}$$

Let $x^*= \hbox {argmin}\{\Vert x\Vert : x \in \hbox {argmin}g\}$ be the element of minimum norm of the nonempty convex closed set $\hbox {argmin}g$. If $\alpha > 0$, then

$$\begin{aligned}\lim _{t\longrightarrow +\infty }\frac{1}{\int _{t_0}^{t}\frac{\epsilon (s)}{s}ds}\int _{t_0}^{t}\frac{\epsilon (s)}{s}\Vert x(s)-x^*\Vert ^2 ds=0 \ \text{ and } \ \liminf _{t\longrightarrow +\infty }\Vert x(t)-x^*\Vert =0.\end{aligned}$$

#### Proof

We introduce the function

$$\begin{aligned}h_{x^*}:[t_0,+\infty )\longrightarrow \mathbb {R},\,h_{x^*}(t)=\frac{1}{2}\Vert x(t)-x^*\Vert ^2.\end{aligned}$$

For every $t\ge t_0$ we have

35 $$\begin{aligned} \ddot{h}_{x^*}(t)+\frac{\alpha }{t}\dot{h}_{x^*}(t)=\Vert \dot{x}(t)\Vert ^2+\left\langle \ddot{x}(t)+\frac{\alpha }{t}\dot{x}(t),x(t)-x^*\right\rangle . \end{aligned}$$

Further, for every $t\ge t_0$, the function $g_t:\mathcal {H}\longrightarrow \mathbb {R},\, g_t(x)=g(x)+\frac{\epsilon (t)}{2}\Vert x\Vert ^2,$ is strongly convex, with modulus $\epsilon (t)$, hence

36 $$\begin{aligned} g_t(x^*)-g_t(x(t))\ge \langle {\nabla }g_t(x(t)), x^*-x(t)\rangle +\frac{\epsilon (t)}{2}\Vert x(t)-x^*\Vert ^2. \end{aligned}$$

But ${\nabla }g_t(x(t))={\nabla }g(x(t))+\epsilon (t) x(t)$ and by using (5) we get

$$\begin{aligned}{\nabla }g_t(x(t))=-\ddot{x}(t)-\frac{\alpha }{t}\dot{x}(t)-\beta {\nabla }^2 g(x(t))\dot{x}(t) \ \text{ for } \text{ every } \ t \ge t_0.\end{aligned}$$

Consequently, (36) becomes

37 $$\begin{aligned} g_t(x^*)-g_t(x(t))\ge \left\langle \ddot{x}(t)+\frac{\alpha }{t}\dot{x}(t)+\beta {\nabla }^2 g(x(t))\dot{x}(t), x(t)-x^*\right\rangle +\frac{\epsilon (t)}{2}\Vert x(t)-x^*\Vert ^2 \ \text{ for } \text{ every } \ t \ge t_0. \end{aligned}$$

By using (35), the latter relation leads to

38 $$\begin{aligned} g_t(x^*)-g_t(x(t))\ge \ddot{h}_{x^*}(t)+\frac{\alpha }{t}\dot{h}_{x^*}(t)+\epsilon (t)h_{x^*}(t)+\langle \beta {\nabla }^2 g(x(t))\dot{x}(t), x(t)-x^*\rangle -\Vert \dot{x}(t)\Vert ^2 \end{aligned}$$

for every $$t \ge t_0$$.

For every $t \ge t_0$, let $x_{\epsilon (t)}$ the unique solution of the strongly convex minimization problem

$$\begin{aligned}\min _{x\in \mathcal {H}}\left( g(x)+\frac{\epsilon (t)}{2}\Vert x\Vert ^2\right) .\end{aligned}$$

Then

$$\begin{aligned}g_t(x^*){-}g_t(x(t))\le g_t(x^*){-}g_t(x_{\epsilon (t)}){=}g(x^*)+\frac{\epsilon (t)}{2}\Vert x^*\Vert ^2-g(x_{\epsilon (t)})-\frac{\epsilon (t)}{2}\Vert x_{\epsilon (t)}\Vert ^2 \le \frac{\epsilon (t)}{2}(\Vert x^*\Vert ^2-\Vert x_{\epsilon (t)}\Vert ^2)\end{aligned}$$

for every $t \ge t_0$ and taking into account (38) we get

39 $$\begin{aligned} \frac{\epsilon (t)}{2}(\Vert x^*\Vert ^2-\Vert x_{\epsilon (t)}\Vert ^2)\ge \ddot{h}_{x^*}(t)+\frac{\alpha }{t}\dot{h}_{x^*}(t)+\epsilon (t)h_{x^*}(t)+\langle \beta {\nabla }^2 g(x(t))\dot{x}(t), x(t)-x^*\rangle -\Vert \dot{x}(t)\Vert ^2 \end{aligned}$$

for every $t \ge t_0$. We have

$$\begin{aligned}\ddot{h}_{x^*}(t)+\frac{\alpha }{t}\dot{h}_{x^*}(t)=\frac{1}{t^\alpha }\frac{d}{dt}\left( t^\alpha \dot{h}_{x^*}(t)\right) \end{aligned}$$

and

$$\begin{aligned}\langle {\nabla }^2 g(x(t))\dot{x}(t), x(t)-x^*\rangle =\frac{d}{dt}\big (\langle {\nabla }g(x(t)), x(t)-x^*\rangle -g(x(t))\big )\end{aligned}$$

hence (39) is equivalent to

40 $$\begin{aligned} \frac{\epsilon (t)}{t}\left( h_{x^*}(t)-\frac{1}{2}(\Vert x^*\Vert ^2-\Vert x_{\epsilon (t)}\Vert ^2)\right) \le \frac{1}{t}\Vert \dot{x}(t)\Vert ^2-\frac{1}{t^{\alpha +1}}\frac{d}{dt}(t^{\alpha }\dot{h}_{x^*}(t))-\frac{\beta }{t}\frac{d}{dt}(\langle {\nabla }g(x(t)), x(t)-x^*\rangle -g(x(t))), \end{aligned}$$

for every $$t \ge t_0$$.

After integrating (40) on $[t_0,t]$, for arbitrary $t > t_0$, it yields

41 $$\begin{aligned} \int _{t_0}^t \frac{\epsilon (s)}{s}\left( h_{x^*}(s)-\frac{1}{2}(\Vert x^*\Vert ^2-\Vert x_{\epsilon (s)}\Vert ^2)\right) ds \le&\ \int _{t_0}^t\left( \frac{1}{s}\Vert \dot{x}(s)\Vert ^2-\frac{1}{s^{\alpha +1}}\frac{d}{ds}\left( s^{\alpha }\dot{h}_{x^*}(s)\right) \right) ds \nonumber \\&\ +\int _{t_0}^t \frac{\beta }{s}\frac{d}{ds}\left( \langle {\nabla }g(x(s)), x^*-x(s)\rangle +g(x(s))\right) ds. \end{aligned}$$

We show that the right-hand side of the above inequality is bounded from above. Indeed, according to Lemma 4.1, one has

$$\begin{aligned}\frac{1}{t}\Vert \dot{x}(t)\Vert ^2\in L^1([t_0,+\infty ),\mathbb {R}),\end{aligned}$$

hence there exists $C_1\ge 0$ such that $\int _{t_0}^t \frac{1}{s}\Vert \dot{x}(s)\Vert ^2\le C_1$ for every $t\ge t_0$. Further, for every $t \ge t_0$,

$$\begin{aligned}\int _{t_0}^t \frac{1}{s^{\alpha +1}}\frac{d}{ds}(s^{\alpha }\dot{h}_{x^*}(s))ds&=\frac{\dot{h}_{x^*}(t)}{t}-\frac{\dot{h}_{x^*}(t_0)}{t_0}+(\alpha +1)\int _{t_0}^t\frac{\dot{h}_{x^*}(s)}{s^2}ds\\&=\frac{\dot{h}_{x^*}(t)}{t}-\frac{\dot{h}_{x^*}(t_0)}{t_0}+(\alpha +1)\left( \frac{{h}_{x^*}(t)}{t^2}-\frac{{h}_{x^*}(t_0)}{t_0^2}\right) +2(\alpha +1)\int _{t_0}^t\frac{{h}_{x^*}(s)}{s^3}ds\\&\ge \frac{\dot{h}_{x^*}(t)}{t}-C_2, \end{aligned}$$

where $$C_2=\frac{\dot{h}_{x^*}(t_0)}{t_0}+(\alpha +1)\frac{{h}_{x^*}(t_0)}{t_0^2}.$$ Consequently,

42 $$\begin{aligned} \int _{t_0}^t \frac{\epsilon (s)}{s}\left( h_{x^*}(s)-\frac{1}{2}(\Vert x^*\Vert ^2-\Vert x_{\epsilon (s)}\Vert ^2)\right) ds \le&\ C_1+C_2-\frac{\dot{h}_{x^*}(t)}{t} \nonumber \\&+\int _{t_0}^t \frac{\beta }{s}\frac{d}{ds}(\langle {\nabla }g(x(s)), x^*-x(s)\rangle +g(x(s)))ds, \end{aligned}$$

for every $t \ge t_0$. According to Lemma 4.1, there exists $C_3$ such that $\frac{1}{t}|\dot{h}_{x^*}(t)|\le C_3 \text{ for } \text{ all } t\ge t_0$, which combined with (42) guarantees the existence of $C_4\ge 0$ such that

43 $$\begin{aligned} \int _{t_0}^t \frac{\epsilon (s)}{s}\left( h_{x^*}(s)-\frac{1}{2}(\Vert x^*\Vert ^2-\Vert x_{\epsilon (s)}\Vert ^2)\right) ds\le C_4+\int _{t_0}^t \frac{\beta }{s}\frac{d}{ds}\left( \langle {\nabla }g(x(s)), x^*-x(s)\rangle +g(x(s))\right) ds \end{aligned}$$

for every $$t\ge t_0$$.

On the other hand, for every $t \ge t_0$,

$$\begin{aligned} \int _{t_0}^t \frac{\beta }{s}\frac{d}{ds}(\langle {\nabla }g(x(s)), x^*-x(s)\rangle +g(x(s)))ds=&\int _{t_0}^t \frac{\beta }{s^2}\big (\langle {\nabla }g(x(s)), x^*-x(s)\rangle +g(x(s))\big )ds \\&+ \frac{\beta }{t}\big (\langle {\nabla }g(x(t)), x^*-x(t)\rangle +g(x(t))\big )\\&-\frac{\beta }{t_0}\big (\langle {\nabla }g(x(t_0)), x^*-x(t_0)\rangle +g(x(t_0))\big ). \end{aligned}$$

From the gradient inequality of the convex function *g* we have

$$\begin{aligned}\langle {\nabla }g(x(t)), x^*-x(t)\rangle +g(x(t))\le g(x^*),\end{aligned}$$

hence

44 $$\begin{aligned} \int _{t_0}^t \frac{\beta }{s}\frac{d}{ds}(\langle {\nabla }g(x(s)), x^*-x(s)\rangle +g(x(s)))ds&\ \le \frac{\beta }{t}g(x^*) +\int _{t_0}^t \frac{\beta }{s^2}g(x^*)ds \nonumber \\&\quad -\frac{\beta }{t_0}(\langle {\nabla }g(x(t_0)), x^*-x(t_0)\rangle +g(x(t_0))), \end{aligned}$$

for all $t \ge t_0$. Obviously the right-hand side of (44) is bounded from above, hence there exists $C_5>0$ such that

45 $$\begin{aligned} \int _{t_0}^t \frac{\beta }{s}\frac{d}{ds}(\langle {\nabla }g(x(s)), x^*-x(s)\rangle +g(x(s)))ds&\le C_5 \text{ for } \text{ every } t\ge t_0. \end{aligned}$$

Combining (43) and (45) we obtain that there exists $C>0$ such that

46 $$\begin{aligned} \int _{t_0}^t \frac{\epsilon (s)}{s}\left( h_{x^*}(s)-\frac{1}{2}(\Vert x^*\Vert ^2-\Vert x_{\epsilon (s)}\Vert ^2)\right) ds&\le C \text{ for } \text{ every } t\ge t_0. \end{aligned}$$

Since $\lim _{t\longrightarrow +\infty }\epsilon (t)=0$ we have $\lim _{t\longrightarrow +\infty }x_{\epsilon (t)}=x^*$, hence $\lim _{t\longrightarrow +\infty }(\Vert x^*\Vert ^2-\Vert x_{\epsilon (t)}\Vert ^2)=0.$ Consequently, by using the l’Hospital rule and the fact that $\int _{t_0}^{+\infty } \frac{\epsilon (t)}{t}dt=+\infty$, we get

$$\begin{aligned}&\lim _{t\longrightarrow +\infty }\!\frac{1}{\int _{t_0}^{t}\frac{\epsilon (s)}{s}ds}\int _{t_0}^{t}\frac{\epsilon (s)}{s}(\Vert x^*\Vert ^2-\Vert x_{\epsilon (s)}\Vert ^2)ds=\!\!\lim _{t\longrightarrow +\infty }\!\frac{\frac{\epsilon (t)}{t}(\Vert x^*\Vert ^2-\Vert x_{\epsilon (t)}\Vert ^2)}{\frac{\epsilon (t)}{t}} \\&\qquad \qquad =\!\!\lim _{t\longrightarrow +\infty }(\Vert x^*\Vert ^2-\Vert x_{\epsilon (t)}\Vert ^2)=0. \end{aligned}$$

Dividing (46) by $\int _{t_0}^t \frac{\epsilon (s)}{s}ds$ and taking into account that $\int _{t_0}^{+\infty } \frac{\epsilon (t)}{t}dt=+\infty$, we obtain that

$$\begin{aligned}\lim _{t\longrightarrow +\infty }\frac{1}{\int _{t_0}^{t}\frac{\epsilon (s)}{s}ds}\int _{t_0}^{t}\frac{\epsilon (s)}{s}\Vert x(s)-x^*\Vert ^2 ds=0.\end{aligned}$$

The last equality immediately implies that

$$\begin{aligned}\liminf _{t\longrightarrow +\infty }\Vert x(t)-x^*\Vert =0.\end{aligned}$$

$$\square $$

#### Remark 4.3

The strong ergodic convergence obtained in [8] for the dynamical system (4) is extended to the dynamical system with Hessian driven damping and Tikhonov regularization term (5) under the same hypotheses concerning the Tikhonov parametrization $t \mapsto \epsilon (t)$.

### Strong convergence

In order to prove strong convergence for the trajectory generated by the dynamical system (5) to an element of minimum norm of $\hbox {argmin}g$ we have to strengthen the conditions on the Tikhonov parametrization. This is done in the following result.

#### Theorem 4.4

Let be $\alpha \ge 3$ and *x* the unique global $C^2$-solution of (5). Assume that

$$\begin{aligned}\int _{t_0}^{+\infty } \frac{\epsilon (t)}{t}dt<+\infty \ \text{ and } \ \lim _{t \longrightarrow +\infty } \frac{\beta }{\epsilon (t)t^{{\frac{\alpha }{3}+1}}}\int _{t_0}^t \epsilon ^2(s)s^{\frac{\alpha }{3}+1}ds=0,\end{aligned}$$

and that there exist $a>1$ and $t_1\ge t_0$ such that

$$\begin{aligned}\dot{\epsilon }(t)\le -\frac{a\beta }{2}\epsilon ^2(t) \ \text{ for } \text{ every } t\ge t_1.\end{aligned}$$

In addition, assume that

- in case $$\alpha =3$$: $$\lim _{t\longrightarrow +\infty }t^2\epsilon (t) =+\infty $$;
- in case $\alpha >3$: there exists $c>0$ such that $t^2\epsilon (t)\ge \frac{2}{3} \alpha \left( \frac{1}{3}\alpha -1+\beta c^2\right)$ for *t* large enough.

If $x^*= \hbox {argmin}\{\Vert x\Vert : x \in \hbox {argmin}g\}$ is the element of minimum norm of the nonempty convex closed set $\hbox {argmin}g$, then

$$\begin{aligned}\liminf _{t\longrightarrow +\infty }\Vert x(t)-x^*\Vert =0.\end{aligned}$$

In addition,

$$\begin{aligned}\lim _{t\longrightarrow +\infty }\Vert x(t)-x^*\Vert =0,\end{aligned}$$

if there exists $T\ge t_0$ such that the trajectory $\{x(t) :t \ge T\}$ stays either in the ball $B(0, \Vert x^*\Vert )$, or in its complement.

#### Proof

*Case I* Assume that there exists $T \ge t_0$ such that the trajectory $\{x(t) :t \ge T\}$ stays in the complement of the ball $B(0, \Vert x^*\Vert )$.

In other words, $\Vert x(t)\Vert \ge \Vert x^*\Vert$ for every $t \ge T$. For $p\ge 0$, we consider the energy functional

47 $$\begin{aligned} \mathcal{E}_b^p(t)=&\ t^{p+1}(t+\alpha -\beta -\beta p-b-1)(g(x(t))-\min g)+t^{p+2}\frac{\epsilon (t)}{2}(\Vert x(t)\Vert ^2-\Vert x^*\Vert ^2) \nonumber \\&\ +\frac{t^p}{2}\Vert b(x(t)-x^*)+t(\dot{x}(t)+\beta {\nabla }g(x(t)))\Vert ^2 \text{ for } \text{ every } t\ge t_0. \end{aligned}$$

We define $t_2:=\max \left( t_1, 2(\beta +\beta p+b+1-\alpha )\right)$. We have that

48 $$\begin{aligned} \mathcal{E}_b^p(t)&\ge t^{p+1}(t+\alpha -\beta -\beta p-b-1)(g(x(t))-\min g)+t^{p+2}\frac{\epsilon (t)}{2}(\Vert x(t)\Vert ^2-\Vert x^*\Vert ^2) \nonumber \\&\ge t^{p+2}\frac{1}{2}(g(x(t))-\min g)+t^{p+2}\frac{\epsilon (t)}{2}(\Vert x(t)\Vert ^2-\Vert x^*\Vert ^2) \text{ for } \text{ every } t\ge t_2. \end{aligned}$$

For every $t\ge t_0$ consider the strongly convex function

$$\begin{aligned}g_t : \mathcal {H} \longrightarrow \mathbb {R}, \ g_t(x)=\frac{1}{2} g(x)+\frac{\epsilon (t)}{2}\Vert x\Vert ^2,\end{aligned}$$

and denote

$$\begin{aligned}x_{\epsilon (t)}:=\mathop {\hbox {argmin}}\limits _{x\in \mathcal {H}}g_t(x).\end{aligned}$$

Since $x^*$ is the element of minimum norm in $\hbox {argmin}\frac{1}{2} g = \hbox {argmin}g$, it holds$\Vert x_{\epsilon (t)}\Vert \le \Vert x^*\Vert$. Using the gradient inequality we have

$$\begin{aligned}g_t(x)-g_t(x_{\epsilon (t)})\ge \frac{\epsilon (t)}{2}\Vert x-x_{\epsilon (t)}\Vert ^2 \ \text{ for } \text{ every } \ x \in \mathcal {H}.\end{aligned}$$

On the other hand,

$$\begin{aligned}g_t(x_{\epsilon (t)})-g_t(x^*)=\frac{1}{2}(g(x_{\epsilon (t)})-\min g)+\frac{\epsilon (t)}{2}(\Vert x_{\epsilon (t)}\Vert ^2-\Vert x^*\Vert ^2)\ge \frac{\epsilon (t)}{2}(\Vert x_{\epsilon (t)}\Vert ^2-\Vert x^*\Vert ^2).\end{aligned}$$

By adding the last two inequalities we obtain

49 $$\begin{aligned} g_t(x)-g_t(x^*)\ge \frac{\epsilon (t)}{2}(\Vert x-x_{\epsilon (t)}\Vert ^2+\Vert x_{\epsilon (t)}\Vert ^2-\Vert x^*\Vert ^2) \ \text{ for } \text{ every } \ x \in \mathcal {H}. \end{aligned}$$

From (48) and (49) we have that for every $t\ge t_2$ it holds

50 $$\begin{aligned} \mathcal{E}_b^p(t)\ge t^{p+2}(g_t(x(t))-g_t(x^*))\ge \frac{\epsilon (t)}{2}t^{p+2}\big (\Vert x(t)-x_{\epsilon (t)}\Vert ^2+\Vert x_{\epsilon (t)}\Vert ^2-\Vert x^*\Vert ^2\big ). \end{aligned}$$

The next step is to obtain an upper bound for $t \mapsto E_b^p(t)$, and to this end we will evaluate its time derivative. For every $t\ge t_0$ we have

51 $$\begin{aligned} \frac{d}{dt}\mathcal{E}_b^p(t)=&\ t^{p}((p+2)t+(p+1)(\alpha -\beta -\beta p-b-1))(g(x(t))-\min g)\nonumber \\ \nonumber&+t^{p+1}(t+\alpha -\beta -\beta p-b-1)\langle {\nabla }g(x(t)),\dot{x}(t)\rangle \\ \nonumber&+ \left( (p+2)t^{p+1}\frac{\epsilon (t)}{2}+t^{p+2}\frac{\dot{\epsilon }(t)}{2}\right) (\Vert x(t)\Vert ^2-\Vert x^*\Vert ^2)+t^{p+2}\epsilon (t))\langle \dot{x}(t),x(t)\rangle \\ \nonumber&+ \frac{p t^{p-1}}{2}\Vert b(x(t)-x^*)+t(\dot{x}(t)+\beta {\nabla }g(x(t)))\Vert ^2\\&+ t^p\langle (b+1)\dot{x}(t)+\beta {\nabla }g(x(t))+t(\ddot{x}(t)+\beta {\nabla }^2 g(x(t))\dot{x}(t)),b(x(t)-x^*)\nonumber \\&+t(\dot{x}(t)+\beta {\nabla }g(x(t)))\rangle . \end{aligned}$$

By using (5) we have

$$\begin{aligned}\ddot{x}(t)+\beta {\nabla }^2 g(x(t))\dot{x}(t)=-\frac{\alpha }{t}\dot{x}(t)-{\nabla }g(x(t))-\epsilon (t)x(t),\end{aligned}$$

hence

52 $$\begin{aligned}&\langle (b+1)\dot{x}(t)+\beta {\nabla }g(x(t))+t(\ddot{x}(t)+\beta {\nabla }^2 g(x(t))\dot{x}(t)),b(x(t)-x^*)+t(\dot{x}(t)+\beta {\nabla }g(x(t)))\rangle \nonumber \\ \nonumber&\quad = \ \langle (b+1-\alpha )\dot{x}(t)+\beta {\nabla }g(x(t))-t({\nabla }g(x(t))+\epsilon (t)x(t)),b(x(t)-x^*)+t(\dot{x}(t)+\beta {\nabla }g(x(t)))\rangle \\ \nonumber&\quad = \ b(b+1-\alpha )\langle \dot{x}(t),x(t)-x^*\rangle +(b+1-\alpha )t(\Vert \dot{x}(t)\Vert ^2+\langle {\nabla }g(x(t)),\dot{x}(t)\rangle )\\ \nonumber&\quad \quad +\beta b\langle {\nabla }g(x(t),x(t)-x^*\rangle +\beta t\langle {\nabla }g(x(t)),\dot{x}(t)\rangle +\beta ^2 t\Vert {\nabla }g(x(t))\Vert ^2\\ \nonumber&\quad \quad -bt\langle {\nabla }g(x(t))+\epsilon (t)x(t),x(t)-x^*\rangle -t^2\langle {\nabla }g(x(t))+\epsilon (t)x(t),\dot{x}(t)\rangle \!\\&\quad \quad -\beta t^2\langle {\nabla }g(x(t))+\epsilon (t)x(t),{\nabla }g(x(t))\rangle \end{aligned}$$

for every $t \ge t_0$. Further, for every $t \ge t_0$,

53 $$\begin{aligned} \Vert b(x(t)-x^*)+t(\dot{x}(t)+\beta {\nabla }g(x(t)))\Vert ^2=&\ b^2\Vert x(t)-x^*\Vert ^2+2b t\langle \dot{x}(t),x(t)-x^*\rangle \nonumber \\&+2b\beta t\langle {\nabla }g(x(t)),x(t)-x^*\rangle \nonumber \\&+t^2\Vert \dot{x}(t)\Vert ^2+2\beta t^2\langle {\nabla }g(x(t)),\dot{x}(t)\rangle \nonumber \\&+\beta ^2 t^2\Vert {\nabla }g(x(t))\Vert ^2, \end{aligned}$$

which means that (51) becomes

54 $$\begin{aligned} \frac{d}{dt}\mathcal{E}_b^p(t) =&\ t^{p}((p+2)t+(p+1)(\alpha -\beta -\beta p-b-1))(g(x(t))-\min g) \nonumber \\ \nonumber&+ \left( (p+2)t^{p+1}\frac{\epsilon (t)}{2}+t^{p+2}\frac{\dot{\epsilon }(t)}{2}\right) (\Vert x(t)\Vert ^2-\Vert x^*\Vert ^2)+ \frac{b^2 p t^{p-1}}{2}\Vert x(t)-x^*\Vert ^2\\ \nonumber&+\frac{(p+2)\beta ^2 t^{p+1}}{2}\Vert {\nabla }g(x(t))\Vert ^2 +\left( b+1-\alpha +\frac{p}{2}\right) t^{p+1}\Vert \dot{x}(t)\Vert ^2\\ \nonumber&+b(b+1-\alpha +p) t^{p}\langle \dot{x}(t),x(t)-x^*\rangle +b\beta ( p+1) t^{p}\langle {\nabla }g(x(t)),x(t)-x^*\rangle \\&-b t^{p+1}\langle {\nabla }g(x(t))+\epsilon (t)x(t),x(t)-x^*\rangle -\beta t^{p+2}\langle {\nabla }g(x(t))+\epsilon (t)x(t),{\nabla }g(x(t))\rangle . \end{aligned}$$

The gradient inequality for the strongly convex function $x\rightarrow g(x)+\frac{\epsilon (t)}{2}\Vert x\Vert ^2$ gives

$$\begin{aligned}&\langle {\nabla }g(x(t))+\epsilon (t)x(t),x^*-x(t)\rangle +\frac{\epsilon (t)}{2}\Vert x(t)-x^*\Vert ^2\le \left( g(x^*)+\frac{\epsilon (t)}{2}\Vert x^*\Vert ^2\right) \\&\quad -\left( g(x(t))+\frac{\epsilon (t)}{2}\Vert x(t)\Vert ^2\right) ,\end{aligned}$$

hence

$$\begin{aligned} -b t^{p+1}\langle {\nabla }g(x(t))&+\epsilon (t)x(t),x(t)-x^*\rangle \le -b t^{p+1}(g(x(t))-g^*)\\&- b t^{p+1}\frac{\epsilon (t)}{2}(\Vert x(t)\Vert ^2-\Vert x^*\Vert ^2)-b t^{p+1}\frac{\epsilon (t)}{2}\Vert x(t)-x^*\Vert ^2 \end{aligned}$$

for every $t \ge t_0$. Plugging this inequality into (54) gives

55 $$\begin{aligned} \frac{d}{dt}\mathcal{E}_b^p(t) \le&\ t^{p}((p+2-b)t+(p+1)(\alpha -\beta -\beta p-b-1))(g(x(t))-\min g) \nonumber \\ \nonumber&{+} \left( (p+2-b)t^{p+1}\frac{\epsilon (t)}{2}{+}t^{p+2}\frac{\dot{\epsilon }(t)}{2}\right) (\Vert x(t)\Vert ^2-\Vert x^*\Vert ^2){+} \left( \frac{b^2 p t^{p-1}}{2}{-}b t^{p+1}\frac{\epsilon (t)}{2}\right) \Vert x(t)-x^*\Vert ^2\\ \nonumber&+\left( \frac{(p+2)\beta ^2 t^{p+1}}{2}-\beta t^{p+2}\right) \Vert {\nabla }g(x(t))\Vert ^2 +\left( b+1-\alpha +\frac{p}{2}\right) t^{p+1}\Vert \dot{x}(t)\Vert ^2\\ \nonumber&+b(b+1-\alpha +p) t^{p}\langle \dot{x}(t),x(t)-x^*\rangle +b\beta ( p+1) t^{p}\langle {\nabla }g(x(t)),x(t)-x^*\rangle \\&-\beta t^{p+2}\epsilon (t)\langle {\nabla }g(x(t)),x(t)\rangle \end{aligned}$$

for every $t \ge t_0$. Further we have for every $t \ge t_0$

56 $$\begin{aligned} b\beta ( p+1) t^{p}\langle {\nabla }g(x(t)),x(t)-x^*\rangle \le&\ \frac{b\beta (p+1)}{4c^2}t^{p+1}\Vert {\nabla }g(x(t))\Vert ^2+b\beta (p+1)c^2t^{p-1}\Vert x(t)-x^*\Vert ^2 \end{aligned}$$

and

57 $$\begin{aligned} -\beta t^{p+2}\epsilon (t)\langle {\nabla }g(x(t)),x(t)\rangle \le&\ \frac{\beta }{a}t^{p+2}\Vert {\nabla }g(x(t))\Vert ^2+\frac{a\beta }{4}\epsilon ^2(t)t^{p+2}\Vert x(t)\Vert ^2, \end{aligned}$$

where $a>1$ and $c>0$ are the constants which are assumed to exist in the hypotheses of the theorem, whereby in case $\alpha =3$ we will take $c=1$.

Combining (55), (56) and (57) and neglecting the nonpositive terms we derive

58 $$\begin{aligned} \frac{d}{dt}\mathcal{E}_b^p(t) \le&\ t^{p}((p+2-b)t+(p+1)(\alpha -\beta -\beta p-b-1))(g(x(t))-\min g) \nonumber \\ \nonumber&+ \left( (p+2-b)t^{p+1}\frac{\epsilon (t)}{2}+t^{p+2}\frac{\dot{\epsilon }(t)}{2}+\frac{a\beta }{4}\epsilon ^2(t)t^{p+2}\right) \Vert x(t)\Vert ^2\\ \nonumber&-\left( (p+2-b)t^{p+1}\frac{\epsilon (t)}{2}+t^{p+2}\frac{\dot{\epsilon }(t)}{2}\right) \Vert x^*\Vert ^2\\ \nonumber&+ \left( \frac{b^2 p t^{p-1}}{2}+b\beta (p+1)c^2t^{p-1}-b t^{p+1}\frac{\epsilon (t)}{2}\right) \Vert x(t)-x^*\Vert ^2\\ \nonumber&+\left( \frac{(p+2)\beta ^2 t^{p+1}}{2}+\frac{b\beta (p+1)}{4c^2}t^{p+1}-\beta \left( 1-\frac{1}{a}\right) t^{p+2}\right) \Vert {\nabla }g(x(t))\Vert ^2 \\&+\left( b+1-\alpha +\frac{p}{2}\right) t^{p+1}\Vert \dot{x}(t)\Vert ^2+b(b+1-\alpha +p) t^{p}\langle \dot{x}(t),x(t)-x^*\rangle \end{aligned}$$

for every $$t \ge t_0$$.

For the remaining of the proof we choose the parameters appearing in the definition of the energy functional as

$$\begin{aligned}b:=\frac{2}{3}\alpha \ \text{ and } \ p:=\frac{1}{3}(\alpha -3).\end{aligned}$$

Since $\alpha \ge 3$, we have

$$\begin{aligned}p+2-b=1-\frac{\alpha }{3}\le 0,\,b+1+p-\alpha =0 \text{ and } b+1+\frac{p}{2}-\alpha =-\frac{p}{2}\le 0.\end{aligned}$$

Notice that, if $\alpha =3$, then $(p+2-b)t+(p+1)(\alpha -\beta -\beta p-b-1)=-\beta \le 0$ and, if $\alpha >3$, then $p+2-b<0$. This means that there exists $t_3\ge t_2$ such that $(p+2-b)t+(p+1)(\alpha -\beta -\beta p-b-1)<0$ for every $t\ge t_3.$ This implies that the term

$$\begin{aligned}t^{p}((p+2-b)t+(p+1)(\alpha -\beta -\beta p-b-1))(g(x(t))-\min g)\end{aligned}$$

in (58) is nonpositive for every $t\ge t_2$ and therefore we will omit it. Further, using that $\lim _{t\longrightarrow +\infty }t^2\epsilon (t) =+\infty$, if $\alpha =3$, and that $t^2\epsilon (t)\ge \frac{2}{3} \alpha (\frac{1}{3}\alpha -1+\beta c^2)$ for *t* large enough, if $\alpha >3,$ we immediately see that there exists $t_4\ge t_3$ such that

$$\begin{aligned}\frac{b^2 p t^{p-1}}{2}+b\beta (p+1)c^2t^{p-1}-b t^{p+1}\frac{\epsilon (t)}{2}\le 0 \text{ for } \text{ every } t\ge t_3.\end{aligned}$$

Finally, since $a>1$, it is obvious that there exists $t_5\ge t_4$ such that

$$\begin{aligned}\frac{(p+2)\beta ^2 t^{p+1}}{2}+\frac{b\beta (p+1)}{4c^2}t^{p+1}-\beta \left( 1-\frac{1}{a}\right) t^{p+2}\le 0 \text{ for } \text{ every } t\ge t_5.\end{aligned}$$

Thus, (58) yields

59 $$\begin{aligned} \frac{d}{dt}\mathcal{E}_b^p(t) \le&\ \left( (p+2-b)t^{p+1}\frac{\epsilon (t)}{2}+t^{p+2}\frac{\dot{\epsilon }(t)}{2}+\frac{a\beta }{4}\epsilon ^2(t)t^{p+2}\right) \Vert x(t)\Vert ^2 \nonumber \\ \nonumber&-\left( (p+2-b)t^{p+1}\frac{\epsilon (t)}{2}+t^{p+2}\frac{\dot{\epsilon }(t)}{2}\right) \Vert x^*\Vert ^2\\ =&\left( (p+2-b)t^{p+1}\frac{\epsilon (t)}{2}+t^{p+2}\frac{\dot{\epsilon }(t)}{2}+\frac{a\beta }{4}\epsilon ^2(t)t^{p+2}\right) (\Vert x(t)\Vert ^2-\Vert x^*\Vert ^2)+ \frac{a\beta }{4}\epsilon ^2(t)t^{p+2}\Vert x^*\Vert ^2, \end{aligned}$$

for every $t\ge t_5$. By the hypotheses, we have that

$$\begin{aligned}(p+2-b)t^{p+1}\frac{\epsilon (t)}{2}+t^{p+2}\frac{\dot{\epsilon }(t)}{2}+\frac{a\beta }{4}\epsilon ^2(t)t^{p+2}\le 0,\end{aligned}$$

for every $t\ge t_5$ and, taking into account the setting considered in this first case, it follows there exists $t_6\ge t_5$ such that

$$\begin{aligned}\Vert x(t)\Vert ^2-\Vert x^*\Vert ^2\ge 0\end{aligned}$$

for every $t\ge t_6$. Hence, (59) leads to

60 $$\begin{aligned} \frac{d}{dt}\mathcal{E}_b^p(t)&\le \frac{a\beta }{4}\epsilon ^2(t)t^{p+2}\Vert x^*\Vert ^2 \ \text{ for } \text{ every } \ t \ge t_6. \end{aligned}$$

By integrating (60) on the interval $[t_6, t]$, for arbitrary $t \ge t_6$, we get

61 $$\begin{aligned} \mathcal{E}_b^p(t)&\le \mathcal{E}_b^p(t_6)+\frac{a\beta }{4}\Vert x^*\Vert ^2 \int _{t_6}^t \epsilon ^2(s)s^{p+2}dt. \end{aligned}$$

Recall that from (50) we have

$$\begin{aligned}\mathcal{E}_b^p(t)\ge \frac{\epsilon (t)}{2}t^{p+2}(\Vert x(t)-x_{\epsilon (t)}\Vert ^2+\Vert x_{\epsilon (t)}\Vert ^2-\Vert x^*\Vert ^2),\end{aligned}$$

which, combined with (61), gives for every $t\ge t_6$ that

62 $$\begin{aligned} \Vert x(t)-x_{\epsilon (t)}\Vert ^2&\le \Vert x^*\Vert ^2-\Vert x_{\epsilon (t)}\Vert ^2+\frac{2E_b^p(t_6)}{\epsilon (t)t^{\frac{1}{3}\alpha +1}} +\frac{a\beta }{2\epsilon (t)t^{\frac{1}{3}\alpha +1}}\Vert x^*\Vert ^2 \int _{t_6}^t \epsilon ^2(s)s^{\frac{1}{3}\alpha +1}dt. \end{aligned}$$

Using that $\lim _{t\longrightarrow +\infty }\epsilon (t)t^{\frac{1}{3}\alpha +1}=+\infty$, $\lim _{t\longrightarrow +\infty }x_{\epsilon (t)}=x^*$ and taking into account the hypotheses of the theorem, we get that the right-hand side of (62) converges to 0 as $t \longrightarrow +\infty$. This yields

$$\begin{aligned}\lim _{t\longrightarrow +\infty } x(t)=x^*.\end{aligned}$$

*Case II* Assume that there exists $T \ge t_0$ such that the trajectory $\{x(t) :t \ge T\}$ stays in the ball $B(0, \Vert x^*\Vert )$.

In other words, $\Vert x(t)\Vert < \Vert x^*\Vert$ for every $t \ge T$. Since

$$\begin{aligned}\int _{t_0}^{+\infty }\frac{\epsilon (t)}{t}dt<+\infty ,\end{aligned}$$

according to Theorem 3.1, we have

$$\begin{aligned}\lim _{t\longrightarrow +\infty }g(x(t))=\min g.\end{aligned}$$

Consider $\overline{x} \in \mathcal {H}$ a weak sequential cluster point of the trajectory *x*, which exists since the trajectory is bounded. This means that there exists a sequence $(t_n)_{n\in \mathbb {N}} \subseteq [T,+\infty )$ such that $t_n\longrightarrow +\infty$ and $x(t_n)$ converges weakly to $\overline{x}$ as $n \longrightarrow +\infty$.

Since *g* is weakly lower semicontinuous, it holds

$$\begin{aligned}g(\overline{x})\le \liminf _{n\rightarrow +\infty } g(x(t_n))=\min g, \ \text{ thus } \ \overline{x}\in \hbox {argmin}g.\end{aligned}$$

Since the norm is weakly lower semicontinuous, it holds

$$\begin{aligned}\Vert \overline{x}\Vert \le \liminf _{n\rightarrow +\infty }\Vert x(t_n)\Vert \le \Vert x^*\Vert ,\end{aligned}$$

which, by taking into account that $x^*$ is the unique element of minimum norm in $\hbox {argmin}g$, implies $\overline{x}=x^*$. This shows that the whole trajectory *x* converges weakly to $x^*$.

Thus,

$$\begin{aligned}\Vert x^*\Vert \le \liminf _{t\rightarrow +\infty }\Vert x(t)\Vert \le \limsup _{t\rightarrow +\infty }\Vert x(t)\Vert \le \Vert x^*\Vert , \ \text{ hence } \ \lim _{t\longrightarrow +\infty }\Vert x(t)\Vert =\Vert x^*\Vert .\end{aligned}$$

But by taking into account that $x(t)\rightharpoonup x^*$ as $t\longrightarrow +\infty$, we obtain that the convergence is strong, that is

$$\begin{aligned}\lim _{t\longrightarrow +\infty }x(t)=x^*.\end{aligned}$$

*Case III* Assume that for every $T \ge t_0$ there exists $t\ge T$ such that $\Vert x^*\Vert > \Vert x(t)\Vert$ and there exists $s\ge T$ such that $\Vert x^*\Vert \le \Vert x(s)\Vert$.

By the continuity of *x* it follows that there exists a sequence $(t_n)_{n\in \mathbb {N}} \subseteq [t_0,+\infty )$ such that $t_n\longrightarrow +\infty$ as $n \longrightarrow +\infty$ and

$$\begin{aligned}\Vert x(t_n)\Vert =\Vert x^*\Vert \text{ for } \text{ every } n\in \mathbb {N}.\end{aligned}$$

We will show that $x(t_n) \longrightarrow x^*$ as $n\longrightarrow +\infty .$ To this end we consider $\overline{x} \in \mathcal {H}$ a weak sequential cluster point of the sequence $(x(t_n))_{n\in \mathbb {N}}.$ By repeating the arguments used in the previous case (notice that the sequence is bounded) it follows that $(x(t_n))_{n\in \mathbb {N}}$ converges weakly to $x^*$ as $n \longrightarrow +\infty$. Since $\Vert x(t_n)\Vert \longrightarrow \Vert x^*\Vert$ as $n\longrightarrow +\infty$, it yields $\Vert x(t_n)- x^*\Vert \longrightarrow 0$ as $n\longrightarrow +\infty$. This shows that

$$\begin{aligned}\liminf _{t\longrightarrow +\infty }\Vert x(t)-x^*\Vert =0.\end{aligned}$$

$$\square $$

#### Remark 4.5

Theorem 4.4 can be seen as an extension of a result given in [8] for the dynamical system (4) to the dynamical system with Hessian driven damping and Tikhonov regularization term (5). One can notice that for the choice $\beta =0$, which means that the Hessian driven damping is removed, the lower bound we impose for $t \mapsto t^2\epsilon (t)$ in case $\alpha >3$ is less tight than the one considered in [8, Theorem 4.1] for the system (4). As we will see later, this lower bound influences the asymptotic behaviour of the trajectory.

In case $\beta >0$, in order to guarantee that

$$\begin{aligned}\lim _{t \longrightarrow +\infty } \frac{\beta }{\epsilon (t)t^{{\frac{\alpha }{3}+1}}}\int _{t_0}^t \epsilon ^2(s)s^{\frac{\alpha }{3}+1}ds=0,\end{aligned}$$

one just have to additionally assume that

$$\begin{aligned}\int _{t_0}^{+\infty } \epsilon (t)dt<+\infty \end{aligned}$$

and that the function

$$\begin{aligned}t\longrightarrow t^{\frac{1}{3}\alpha +1}\epsilon (t) \ \text{ is } \text{ nondecreasing } \text{ for } \ t \ \text{ large } \text{ enough }.\end{aligned}$$

This follows from Lemma A.1, by also taking into account that $\lim _{t\longrightarrow +\infty }\epsilon (t)t^{\frac{\alpha }{3}+1}=+\infty$.

Combining the main results in the last two sections, one can see that if

$$\begin{aligned}\int _{t_0}^{+\infty } t \epsilon (t)dt<+\infty ,\end{aligned}$$

the function

$$\begin{aligned}t\longrightarrow t^{\frac{1}{3}\alpha +1}\epsilon (t) \ \text{ is } \text{ nondecreasing } \text{ for } \ t \ \text{ large } \text{ enough },\end{aligned}$$

there exist $a>1$ and $t_1\ge t_0$ such that

$$\begin{aligned}\dot{\epsilon }(t)\le -\frac{a\beta }{2}\epsilon ^2(t) \ \text{ for } \text{ every } t\ge t_1,\end{aligned}$$

and

- in case $$\alpha =3$$: $$\lim _{t\longrightarrow +\infty }t^2\epsilon (t) =+\infty $$;
- in case $\alpha >3$: there exists $c>0$ such that $t^2\epsilon (t)\ge \frac{2}{3} \alpha \left( \frac{1}{3}\alpha -1+\beta c^2\right)$ for *t* large enough,

then one obtains both fast convergence of the function values and strong convergence of the trajectory to the minimal norm solution. This is for instance the case when $\epsilon (t) = t^{-\gamma }$ for all $\gamma \in (1,2)$.

**Fig. 2:**
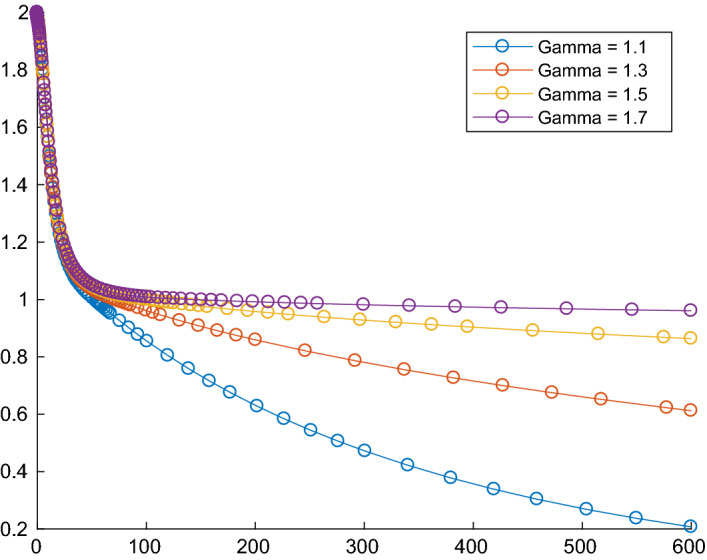
The behaviour of the trajectories generated by the dynamical system (5) in relation with the minimization of the function given in (6) for $\alpha =200$, $\beta =1$, $\epsilon (t)=t^{-\gamma }$ and different values for $\gamma \in (1,2)$

In the following, we would like to comment on the role on the condition in Theorem 4.4 which asks, in case $\alpha >3$, for the existence of a positive constant *c* such that $t^2\epsilon (t)\ge \frac{2}{3} \alpha (\frac{1}{3}\alpha -1+\beta c^2)$ for *t* large enough. To this end it is very helpful to visualize the trajectories generated by the dynamical system (5) in relation with the minimization of the function given in (6) for a fixed large value of $\alpha$ and Tikhonov parametrizations of the form $t \mapsto \epsilon (t)=t^{-\gamma }$, for different values of $\gamma \in (1,2)$. The trajectories in the plot in Fig. 2 have been generated for $\alpha =200$ and $\beta =1$ and are all approaching the minimum norm solution $x^*=0$. The norm of the difference between the trajectory and the minimum norm solution is guaranteed to be bounded from above by a function which converges to zero, after the time point *t* is reached at which the inequality $t^2\epsilon (t)\ge \frac{2}{3} \alpha (\frac{1}{3}\alpha -1+\beta c^2)$ “starts” being fulfilled. For large $\alpha$ and the Tikhonov parametrizations considered in our experiment, the closer $\gamma$ is to 1 is, the faster is this inequality fulfilled. This is reflected by the behaviour of the trajectories plotted in Fig. 2.

Finally, we would like to formulate some possible questions of future research related to the dynamical sytem (5):

- In [7, Theorem 3.4] it has been proved for the dynamical system (1) that, when *g* is strongly convex, the rates of convergence of the function values and the tracjectory are both of $O(t^{-\frac{2}{3}\alpha })$, thus they can be made arbitrarily fast by taking $\alpha$ large. It is natural to ask if similar rates of convergence can be obtained in a similar setting for the dynamical system (5) (see, also, [8, Section 5.4]).
- In the literature, in the context of dynamical systems, regularization terms have been considered not only in open-loop, but also in closed-loop form (see, for instance, [12]). It is an interesting question if one can obtain for the dynamical system (5) similar results if the Tikhonov regularization term is taken in closed-loop form.
- A natural question is to formulate proper numerical algorithms via time discretization of (5), to investigate their theoretical convergence properties, and to validate them with numerical experiments.

## Appendix

In this appendix, we collect some lemmas and technical results which we will use in the analysis of the dynamical system (5). The following lemma was stated for instance in [8, Lemma A.3] and is used to prove the convergence of the objective function along the trajectory to its minimal value.

### Lemma A.1

Let $\delta >0$ and $f\in L^1((\delta ,+\infty ),\mathbb {R})$ be a nonnegative and continuous function. Let $\varphi :[\delta ,+\infty )\longrightarrow [0,+\infty )$ be a nondecreasing function such that $\lim _{t\longrightarrow +\infty }\varphi (t)=+\infty .$ Then it holds

$$\begin{aligned}\lim _{t\longrightarrow +\infty }\frac{1}{\varphi (t)}\int _\delta ^t\varphi (s)f(s)ds=0.\end{aligned}$$

The following statement is the continuous counterpart of a convergence result of quasi-Fejér monotone sequences. For its proofs we refer to [1, Lemma 5.1].

### Lemma A.2

Suppose that $F:[t_0,+\infty )\rightarrow \mathbb {R}$ is locally absolutely continuous and bounded from below and that there exists $G\in L^1([t_0,+\infty ), \mathbb {R})$ such that

$$\begin{aligned} \frac{d}{dt}F(t)\le G(t) \end{aligned}$$

for almost every $t \in [t_0,+\infty )$. Then there exists $\lim _{t\longrightarrow +\infty } F(t)\in \mathbb {R}$.

The following technical result is [11, Lemma 2].

### Lemma 4.6

Let $u : [t_0,+\infty )\longrightarrow \mathcal {H}$ be a continuously differentiable function satisfying $u(t) +\frac{t}{\alpha }\dot{u}(t)\longrightarrow u \in \mathcal {H}$ as $t\longrightarrow +\infty ,$ where $\alpha >0.$ Then $u(t)\longrightarrow u$ as $t \longrightarrow +\infty .$

The continuous version of the Opial Lemma (see [7]) is the main tool for proving weak convergence for the generated trajectory.

### Lemma A.3

Let $S \subseteq \mathcal {H}$ be a nonempty set and $x : [t_0, +\infty ) \rightarrow H$ a given map such that:

$$\begin{aligned}&\mathrm{(i)} \quad \text{ for } \text{ every } z \in S \ \text{ the } \text{ limit } \ \lim \limits _{t \longrightarrow +\infty } \Vert x(t) - z \Vert \ \text{ exists };\\&\mathrm{(ii)} \quad \text {every weak sequential limit point of } x(t) \text { belongs to the set }S. \end{aligned}$$

Then the trajectory *x*(*t*) converges weakly to an element in *S* as $t \rightarrow + \infty$.
